# Binomial models uncover biological variation during feature selection of droplet-based single-cell RNA sequencing

**DOI:** 10.1371/journal.pcbi.1012386

**Published:** 2024-09-06

**Authors:** Breanne Sparta, Timothy Hamilton, Gunalan Natesan, Samuel D. Aragones, Eric J. Deeds

**Affiliations:** 1 Department of Integrative Biology and Physiology, University of California, Los Angeles, California, United States of America; 2 Institute for Quantitative and Computational Biosciences, University of California, Los Angeles, California, United States of America; 3 Bioinformatics Interdepartmental Program, University of California, Los Angeles, California, United States of America; Johns Hopkins University Whiting School of Engineering, UNITED STATES OF AMERICA

## Abstract

Effective analysis of single-cell RNA sequencing (scRNA-seq) data requires a rigorous distinction between technical noise and biological variation. In this work, we propose a simple feature selection model, termed “Differentially Distributed Genes” or DDGs, where a binomial sampling process for each mRNA species produces a null model of technical variation. Using scRNA-seq data where cell identities have been established *a priori*, we find that the DDG model of biological variation outperforms existing methods. We demonstrate that DDGs distinguish a validated set of real biologically varying genes, minimize neighborhood distortion, and enable accurate partitioning of cells into their established cell-type groups.

## Author summary

Single-cell omics technologies measure tens of thousands of genes in up to millions of individual cells. Yet, the sheer dimensionality of the data poses a challenge to its intelligibility. A typical first step in reducing the dimensionality is to apply a feature selection model that distinguishes real biological signals from technical noise. Yet without an appropriate model of technical noise, feature selection can introduce bias into the downstream analysis of the data. In this work, we demonstrate that, in the analysis of single-cell RNA sequencing data, the standard approach of finding Highly Variable Genes (HVGs) induces severe distortion and bias into the analysis of data, when compared to true biological variation that is known *a priori*. To address this issue, we present a new feature selection model and demonstrate that our model outperforms existing methods in its ability to accurately identify real biological variation.

## Introduction

Single-cell RNA sequencing has advanced the resolution at which variation in gene expression can be observed [1]. Recent improvements in scRNA-seq technology have enabled the measurement of tens of thousands of genes across hundreds of thousands to millions of cells [2]. Yet, interpreting transcriptional variation in these extremely high-dimensional datasets remains a challenge at the forefront of biological research [3].

In addition to the high levels of biological variation observed in scRNA-seq data, the sparsity of the data can pose challenges during downstream analysis procedures [4]. There exist very small quantities of mRNA inside of single cells, and even with advanced microfluidic technologies, the capture probability for any given mRNA is low [5]. As a result, a typical scRNA-seq experiment can generate a gene-by-cell expression matrix where ~95% of the entries are zeros. The observance of this large fraction of zeros, colloquially termed “drop-outs”, has left an impression that scRNA-seq data is zero inflated [6–9]. However, there is a growing body of empirical and theoretical work that demonstrates that, given the distributions of gene-count data, we do not observe more zeros in scRNA-seq data than would be expected based on a sampling process where the probability of capturing any given mRNA molecule is low [10–12].

A major contributor to this observation is the use of technologies in droplet-based scRNA-seq approaches that reduce technical error [13–16]. In these experiments, individual mRNA’s from lysed cells are bound to Unique Molecular Identifiers, or UMI’s, prior to PCR-based amplification, thus overcoming amplification bias when measuring mRNA abundance [17]. Despite these advances, scRNA-seq approaches still entail a low capture probability for individual mRNA molecules, and the resulting sparsity of this high-dimensional data pose challenges for the analysis of biological variation [3]. Standard approaches often employ a feature selection step, where observed variation in counts of mRNA species across cells is compared to a model of measurement noise [18,19]. The aim of this feature selection step is to identify genes whose expression levels vary due to meaningful biological differences in the population, thus varying more than what would be expected due to technical noise alone. This step is often applied prior to cell-type clustering, such that clustering is performed on only the set of genes that are expected to be biologically informative.

While many null models of biological variation have been proposed, which feature selection method is most appropriate remains unresolved [19–22]. The most popular approach involves finding “Highly Variable Genes” (or HVGs), which identifies genes with higher variance than what would be expected given the average UMI count for each gene [23–25]. Yet, the procedure itself introduces bias into variance estimates, through a series of nonlinear transformations that are performed prior to the HVG selection step [10]. In the standard analysis pipeline, raw UMI counts are first transformed with a counts-per-million (CPM) normalization and then with a log+1 transformation. These transformations are motivated by the ideas of normalizing for cell-size factors and stabilizing the variance for genes whose averages are orders of magnitudes different, respectively. However, both transformations have the undesirable effect of increasing the distance between 0 and non-zero values within a gene’s distribution [10,17,26]. As a result, these transformations artificially increase the variance of genes, and genes with mean counts that are closer to zero are disproportionality inflated [10]. Further, CPM normalization aims to adjust counts for potential read depth-differences that occur from differences in cell size. However, in droplet-based approaches, the assumption that cell-size can affect read-depth lacks empirical support. As such, the utility of the HVG procedure has not been well-established, and can potentially skew downstream cell-clustering and analysis results.

As a result, there is an ongoing effort to improve feature selection in scRNA-seq. One common alternative is to model the abundance of zeros, or ‘dropouts’, in gene distributions and to identify features that have more zeros than expected. For instance, a recent software package developed by Andrews and Hemberg offers three different null models that develop an expectation for the relationship between the ‘dropout’ rate and the mean expression level of each gene: M3Drop, NBDrop, and NBDisp. In M3Drop, a dropout rate parameter is fit to the whole transcriptome, and a Michaelis-Menten-style hyperbolic function is used to identify outliers where the gene-specific dropout rate exceeds the population expectation [19]. NBDrop uses a negative binomial distribution to model the fraction of zeros per gene as a function of the mean gene count that is adjusted for the sequencing efficiency, or total number of counts, for each cell. NBDisp uses the same negative binomial model as NBDrop, but is similar to the HVG method in that it uses linear regression to model the relationship between the mean and the estimated dispersion to identify a set of overly-dispersed genes. In another method similar to the NBDrop approach, Townes and co-wokers identified a set of genes with a greater fraction of zeros than is expected, using a multinomial model where the probability of a mRNA being captured depends on its relative abundance within each cell [10].

In each of these previous studies, feature selection models are evaluated based on clustering performance using biological data with “ground truth” cell identities, or by their ability to recover differentially expressed genes that have been identified using bulk RNA-seq methods. Using these approaches, it has been demonstrated that the HVG method only marginally improves performance compared to a randomized feature selection control [19]. Yet, the HVG method remains extremely popular [18–23,27–35]. In addition to uncertainty about the ability of various methods to identify an informative set of genes with real biological variation, the HVG, NBDisp, and the Townes method all entail arbitrary decisions about the number of features to use in the downstream clustering procedure. Because the choice of feature selection models can significantly alter a study’s results, there is a need to further develop statistically-grounded feature selection approaches, and to subject these methods to rigorous tests that evaluate their capacity to identify genes that exhibit *bona fide* biological variation within a population.

In this work, we proposed a simple binomial model that can be used to identify differentially distributed genes, or DDGs. In this model, each mRNA has the same probability of being captured during the initial phase of the scRNA-seq experiment, which results in a binomial sampling process of the mRNA molecules within the cell. We then consider the simple null model where there is no biological variation in a gene’s expression across the population, and then calculate the probability that purely technical variation due to the mRNA capture process could generate the observed expression patterns. While similar in spirit to the multinomial model developed by Townes [10], our inclusion of a specific null model allows us to calculate a *p*-value that represents the chance that the observed variation in a gene’s expression could be explained purely by technical noise. As a result, our approach allows researchers to define a standard False Discovery Rate (FDR) that sets the number of false positives they are willing to accept, rather than an arbitrary cutoff in the number of genes to consider for further analysis.

We validated this model using a standard synthetic cDNA spike-in scRNA-seq data set, where all of the variation should arise from the sampling process. We found that the vast majority of these genes were consistent with the null model, as expected. We further tested our approach by clustering cells from a study of FACS-sorted lymphocytes, where cell identity markers are obtained prior to scRNA-seq [15]. We compared our model to a number of existing feature selection approaches, and found that DDGs better retain the structure of variation among established cell identities and more accurately identified genes that are differentially expressed between cell types. DDGs also provide a feature set that better partitions groups of cells in accordance with their established cell identity labels. We found that, while feature selection only marginally improves cell clustering performance compared to the full feature set, the DDG approach enables dimensionality reduction without loss of cell neighborhood structures. Overall, our findings suggest that, compared to existing methods, DDGs can more comprehensively identify genes whose expression patterns demonstrate bona fide biological variation.

## Results

### Characterizing patterns of gene expression variation across tissues and multicellular organisms

Single-cell RNA sequencing technology has promised to map functional diversity by quantifying global gene expression patterns at the resolution of individual cells. This approach has the potential to revolutionize our understanding of how gene expression, cellular identities, and tissue function are related. Yet elucidating the molecular organization of tissue function remains an ongoing challenge. In scRNA-seq experiments, the dominant approach is to identify cell-type specific changes in gene expression across varying experimental contexts. Here, it is hypothesized that clustering the transcriptome can produce a model of cell types, where cells of the same type are expected to express similar sets of genes and have similar functions [23,36,37]. To gain intuition about the structure of transcriptional variation, we took the inverse approach, and sought to characterize groups of genes that are expressed across similar sets of cells. To identify and compare communities of genes that are expressed in similar patterns across complex tissues, we performed gene-clustering across a diverse set of scRNA-seq data sets.

**Fig 1:**
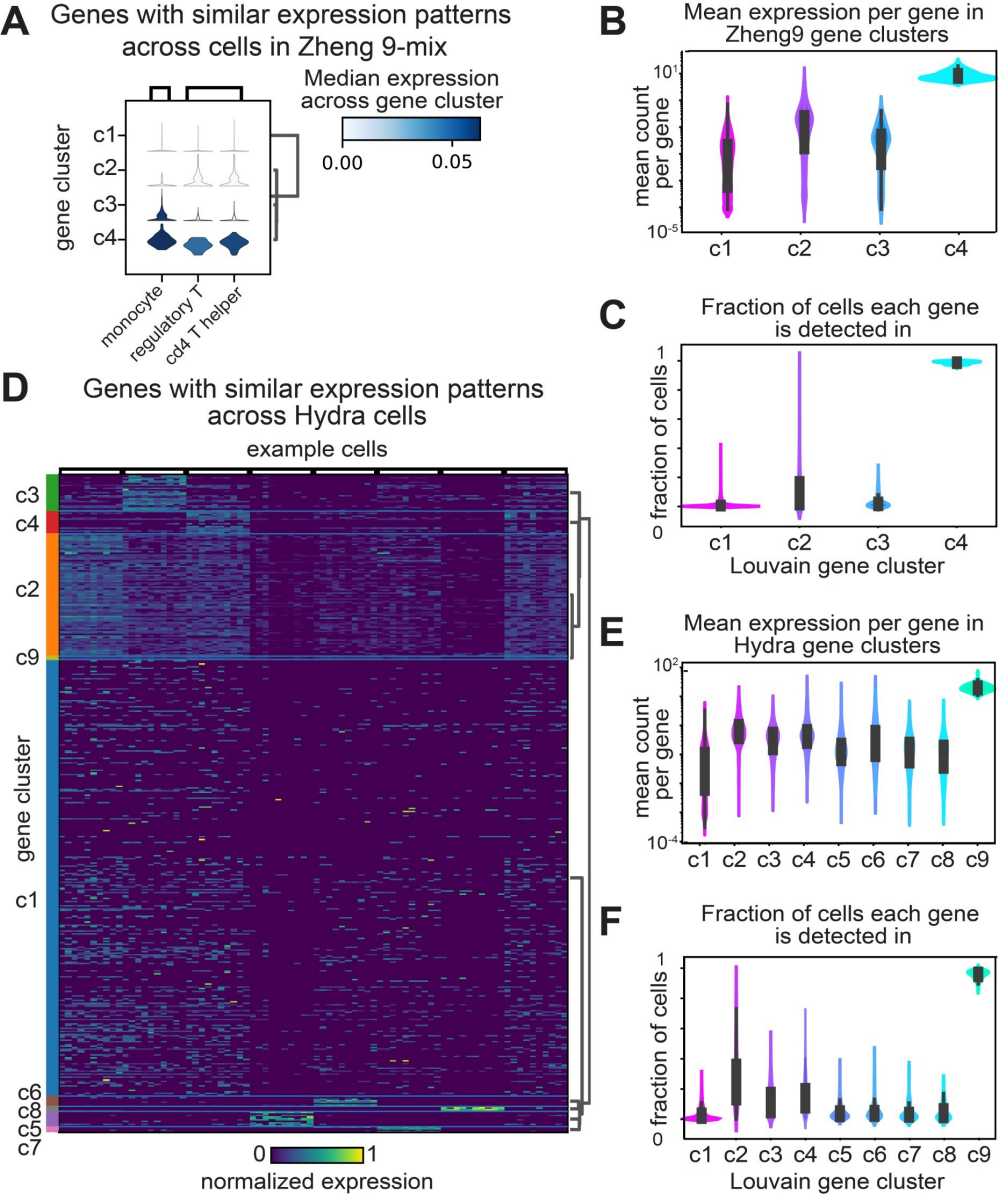
Gene clustering reveals patterns of expression variation across complex tissues and whole organisms. **A)** Violin plots depicting the estimated probability density for gene clusters in Zheng-9 lymphocytes. Each column represents a particular cell, and the violin plot is colored by the average expression level across the set of genes within the gene cluster. **B)** Kernel density estimation for the distribution of average gene expression across cells for each gene cluster group in the Zheng-9 lymphocytes. **C)** Kernel density estimation for the distribution of fraction of cells each gene in the gene cluster is identified in, for the Zheng-9 lymphocytes. **D)** Heat map depicting the normalized expression level of genes grouped by gene cluster membership in the Hydra. Each row represents a gene and column represents a particular cell, and the entry is colored by the normalized expression level. **E)** Kernel density estimation for the distribution of average gene expression across cells for each gene cluster group in the Hydra. **F)** Kernel density estimation for the distribution of fraction of cells each gene in the gene cluster is identified in for the Hydra.

Louvain clustering was performed on the genes of scRNA-seq data collected from human lymphocytes, mouse bladder, mouse kidney, hydra, or planaria (Figs 1 and S1) [15,27,28,30,35,38]. Across these samples we observed three general expression patterns: 1) A group of genes that are nearly ubiquitously expressed across all cells in the sample. These genes are comprised largely of ribosomal proteins, and genes involved in energy production, such as the ATP-synthase. 2) Sparsely expressed genes that are found in few, non-overlapping sets of cells, with no apparent structure in the expression pattern. 3) Groups of genes that are differentially expressed in distinct groups of cells (Fig 1).

We hypothesized that genes expressed in the third, differentially expressed group are involved in the production of distinct cellular identities. In general, the physiological diversity of the sample correlated to the number of gene clusters that are expressed in distinct groups of cells. For example, in the Zheng-9 lymphocyte data we observed one cluster of ubiquitously expressed genes (cluster 4), one cluster of sparsely expressed genes (cluster 1), and two clusters of differentially expressed genes (cluster 2 & 3) (Fig 1A, 1B and 1C). In contrast, in the Hydra data, we found six differentially expressed clusters (Fig 1D, 1E and 1F).

Across our gene clusters, we observed a trend between the mean expression level per gene and the fraction of cells in which each gene was identified (Figs 1B, 1C, 1E, 1F and S1). Genes with high mean expression levels are observed in nearly every sequenced cell. In contrast, of all the gene groups, the subset of genes which appear to be expressed at random have the lowest mean expression levels. Groups of genes that are differentially expressed have expression levels that fall between these two extremes. This observed trend between the mean expression level of a gene, and the number of cells that gene is observed within, suggests methods that relate these two metrics may be useful in identifying genes that capture real biological variation.

### A binomial model of mRNA capture identifies genes expressed in fewer cells than expected

For the analysis of scRNA-seq data, many procedures for identifying biologically varying genes have been developed. In the standard HVG feature selection approach, researchers model the mean-variance relationship (dispersion) of transformed gene count data and select those genes that are the most variable for downstream cell-type clustering. The HVG procedure depends on the assumption that variance is proportional to the mean, across the span of mean values observed in the data. Yet, in general, the mean-variance relationship depends on the distribution the sample was drawn from, and in scRNA-seq data, the distribution of particular gene expression patterns across cells is not known. Further, the variance of a gene may be underestimated when the mean counts are close to zero [39]. To satisfy the assumptions of the HVG model and enable comparison of dispersion across genes whose means span orders of magnitude of values, a log+1 transformation is applied to the normalized count-by-cell matrix. However, rather than achieving a variance-stabilizing effect, this transformation disproportionately increases the dispersion of genes where a greater fraction of the gene counts per cell are zeros. As a result, the HVG approach has demonstrated the capacity to enrich for a set of genes biased to have low expression values [10]. Whether HVGs are biologically varying genes, and whether HVGs are operationally useful for cell-type clustering, has not been systematically characterized.

Other existing models assert different assumptions about the structure of gene expression and the capture process of scRNA-seq technology. The M3Drop, NBDrop, NBDisp, and Townes models all make subtly different assumptions about the relationships between the fraction of zeros per gene and the size of a cell. In the M3Drop method, the drop-out process is modeled as a kinetic process, such that there exists error around the mean counts per gene [19]. In the NBDrop and NBDisp method, the relationship between the fraction of zeros and mean gene count is modified by the total number of reads across each cell the gene is observed in [19]. These models assume that the capture efficiency changes depending on the size of a cell. Similarly, the Townes method models the mRNA capture step as a process where there is competition to be counted, essentially positing that there is a fixed number of mRNA counts per cell [10].

**Fig 2:**
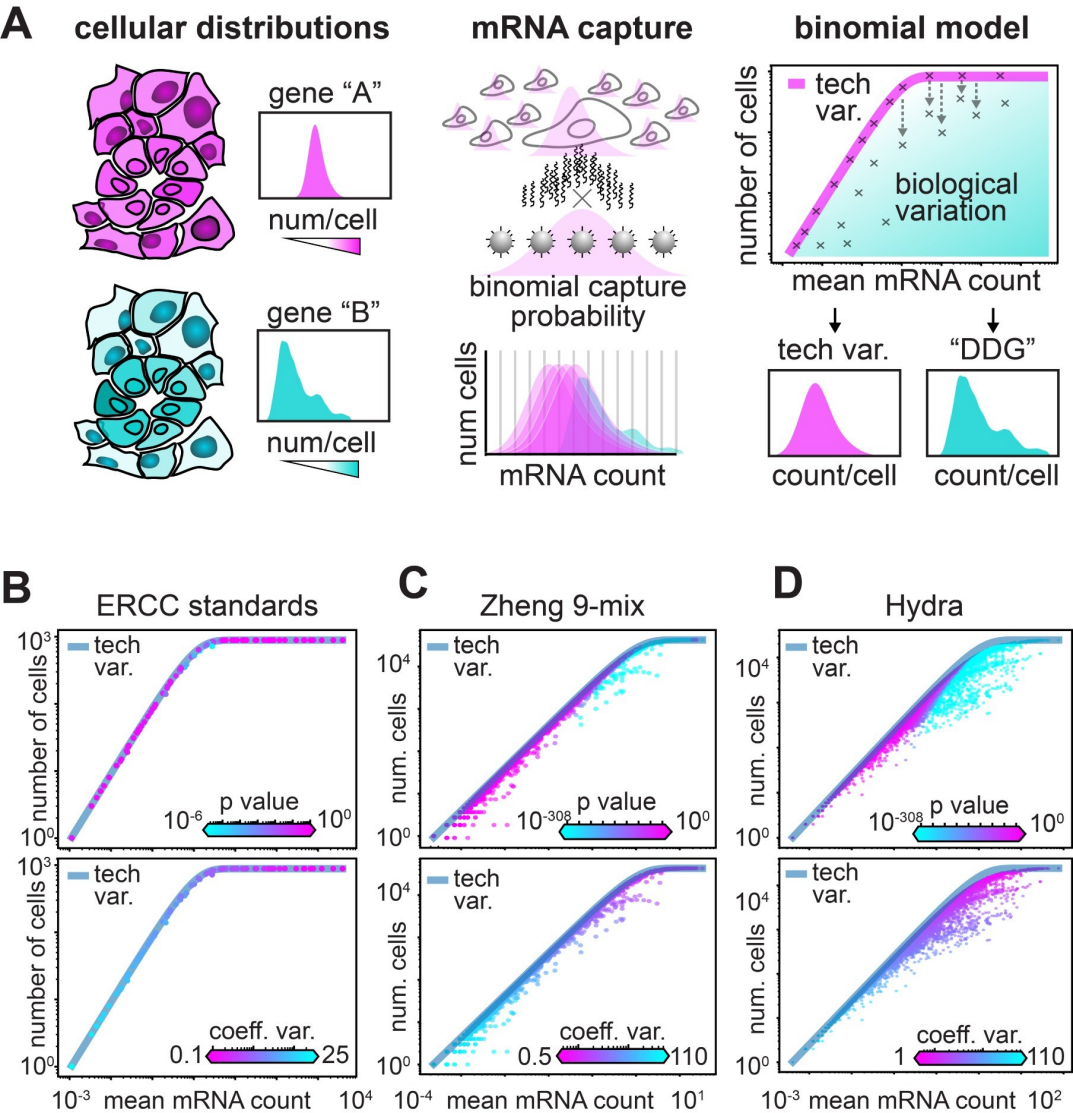
A binomial model of mRNA capture identifies genes expressed in fewer cells than expected. **A)** Schematic of the null model for biologically varying genes. The first panel illustrates uniformly (pink) and differentially (cyan) distributed gene expression across a sample of cells, with illustrated histograms of each gene’s count distribution across the set of cells. The second panel illustrates the mRNA capture as a binomial process, where the probability of capture for each mRNA is stochastic. The third panel depicts the expected relationship of the average mRNA level and the number of cells each gene is observed in, where each ‘x’ on the graph represents a specific gene. The pink line illustrates the expected relationship if the only variation that is observed arises from the binomial sampling process. **B-D)** Scatter of average mRNA count per gene versus the number of cells each gene is identified in for three datasets: B) the synthetic ERCC data, C) the Zheng-9 lymphocytes, and D) the Hydra. In the top panel each gene is colored by the P-value computed from the DDG model, while in the bottom panel each gene is colored by the coefficient of variation.

In this work, we develop a different null model of variation in counts based on a binomial sampling process under simple assumptions. In our model, the observed mean expression level of a particular gene is used to develop an expectation of what fraction of cells we would find each gene in–if the gene was expressed at exactly the same level across cells (Fig 2A). This model does not require assumptions about the underlying distributions of mRNA in cells, nor the mean-variance relationship of gene expression. Based on empirical data, we propose that it is more accurate to model the binding of mRNA to UMI-coated beads as a stochastic process that is not saturated, nor affected by the size of a cell. In our model, every mRNA has an equal probability of binding to a bead, giving a binomial sampling process where each mRNA molecule can be considered as an independent “trial” with a fixed capture probability.

This model is described in detail in the *Methods* section, but we will briefly explain it here. First, consider some gene in the genome, which we will call gene *i*. For any cell *j* in the dataset, we define the observed amount of mRNA in that cell to be *m*_*i*,*j*_ and the *real amount of mRNA* the cell had before the capture process to be *M*_*i*,*j*_. In other words, *M*_*i*,*j*_ is the actual amount of mRNA for gene *i* that was in cell *j*, while *m*_*i*,*j*_ is the amount of counts we end up actually observing for that gene in the experiment. We now imagine that, in each cell in each droplet in the experiment, there is a fixed “capture probability” *p*_*c*_; this is just the chance that any given mRNA molecule is captured on a bead, amplified effectively, etc., and thus is detected as a UMI. We assume that this probability is the same for all genes and for all cells; we will discuss how this parameter is determined, and the sensitivity of our results to the value of this parameter, in detail below. This leads to a simple model where every mRNA in every cell is subjected to a Bernoulli trial, leading to a Binomial sampling process with a total of *M*_*i*,*j*_ trials (Fig 2A).

A key parameter in this model is *M*_*i*,*j*_, the real amount of mRNA that the cell started with, which of course we don’t know. To build a simple null model, we ask what would happen if the observed variation in the data were *entirely* technical. In other words, given Binomial sampling, and the fact that capture probabilities in scRNA-seq experiments are very low [5], there would still be a lot of observed variation in the UMI counts in the data even if every cell in the experiment had exactly the same amount of mRNA to start with. We call this value *M*_*i*_, and note that, under this null model, the *average amount* of UMI counts observed for gene *i* would be *E*(*m*_*i*_) = *M*_*i*_*p*_*c*_. Since we can easily calculate the empirical average of the observed UMI counts for any gene, we can estimate $M_{i}=\frac{E(M_{i})}{p_{c}}$. In other words, to obtain an estimate for this constant value of mRNA in each cell, we just take the observed average expression level and divide it by the capture probability.

This model suggests a natural relationship between two key quantities that we can easily calculate from the experiment: the expected number of cells that express a given gene (i.e. have at least one UMI for that gene) and the average expression level of that gene. Call the number of cells that express gene *i N*_*c*,*i*_. Then the expected value for this number is:

$$E(N_{c,i})=N_{T}\left(1-{\left(1-p_{c}\right)}^{\frac{E(m_{i})}{p_{c}}}\right),$$

where *N*_*T*_ is the total number of cells in the experiment. Note that the portion of this expression in parentheses is just the probability of having at least 1 successful capture event in the experiment.

One major difference between our proposed model and the model developed by Townes and co-workers [10] is in how cell-size factors are handled. In our null model, every cell starts with an identical amount of mRNA for gene *i*, and cell-size effects can still contribute to biological variation. In other words, variation in the population that arises from cases where a cell is larger, and thus has more mRNA for gene *i* than a cell that is smaller, will deviate from the predictions of our null model. In the Townes method, the probability of capture for each mRNA is capped by its proportional expression in each cell, and natural variation in total mRNA’s per cell cannot be accounted for.

To test the relationship between mean expression level and number of cells expressing a given gene, we used scRNA-seq data created from a sample where no biological variation exists. In this experiment, scRNA-seq was performed on droplets spiked with a set of standard, synthetic ERCC control cDNA, and lacking any biological cells [15]. We observed that, in the absence of real biological variation, the vast majority of the spiked cDNA species fell along the expected relationship predicted by a binomial sampling process (i.e. the equation above, Fig 2B). In addition to simply predicting the relationship between mean mRNA expression and the total number of cells in which a gene is observed, we can use our model to calculate the *probability* that we would observe *N*_*c*,*i*_ for a given gene *i* given it’s observed mean expression level *E*(*m*_*i*_) (see Methods). This gives us a natural way to define a *p*-value for the null model, which is the probability of obtaining the observed number (or fewer) cells expressing that gene, given *E*(*m*_*i*_). We term genes for which the observed expression pattern yields significant *p*-values to be “Differentially Distributed Genes” or DDGs, since the variation in the expression pattern for that gene deviates significantly from what we would expect if the gene were expressed identically across all the cells. We use the standard Benjamini-Hochberg procedure to correct for multiple hypothesis testing. Interestingly, if we set the False Discovery Rate (FDR) to 1%, only 5 of the 93 ERCC spike-ins are significant (Fig 2B), suggesting that the bulk extent of technical variation in droplet-based scRNA-seq can be explained by a binomial sampling process. The FDR can be modified in order to make the threshold for inclusion of a gene in the DDG set more or less stringent.

Given a binomial sampling process for mRNA capture, we can expect the fraction of mRNA’s that are actually detected in an experiment to vary depending on the probability of mRNA capture. In droplet-based scRNA-seq, this capture probability is expected to be between 5–10% [5], but can be difficult to estimate for any given experiment in the absence of appropriate controls. Since the DDG model requires specifying a parameter value for_,_ the capture probability, *p*_*c*_, we next evaluated our model’s robustness to this parameter choice. To do this, we created a Gaussian Mixture Model (GMM) with 3 cell types, and 3000 cells per cell type. There are also a total of 1000 genes for each cell. If a gene is chosen as a “marker” for a given cell type, then for all the cells of that type, the counts of that gene are drawn from a Gaussian with a mean of 35 and a standard deviation of 2. If a gene is not a marker gene, then the count value is drawn from a Gaussian with a mean of 15 and a standard deviation of 1. Note that these values were taken from rough estimates of useful “marker genes” in datasets where we know the cell type identities *a priori* (see Methods). As a result, there are a total of 900 marker genes, 300 genes being markers for each of the 3 cell types. These should be “true” DDGs. There are also 100 genes that are not markers for anything, and serve as negative controls.

We then simulated a binomial sampling process by performing a Bernoulli trial for each “UMI count” in the data, varying the capture probability from 1% up to 50%. We find that under reasonable parameter regimes where some noise exists, but not enough to wash out the signal entirely, the DDG model is able to recover 100% of the ground truth genes (S2C and S2F Fig). This is true even when the DDG model parameter for capture probability is set to 5%, but the true capture probability in the simulation ranges from 2%-20%. This suggests that the DDG model is fairly robust to misspecification of the capture probability, at least within a reasonable range. Another interesting result of this GMM is that the model never mis-identified a “non-marker” gene as a DDG. This indicates that, even though our null model assumes that every cell has *exactly the same* number of mRNAs, small variation in the initial starting number of mRNAs does not interfere with the identification of genes that vary in a significant way biologically. Interestingly, the HVG approach was not able to recover the full set of ground truth marker genes for this simulated dataset, suggesting the HVG approach may struggle to find genes with *bona fide* significant differences across cell types (S2F Fig).

We next applied our DDG model to scRNA-seq data with real biological variation, using data collected from the Zheng-9 lymphocytes as well as the Hydra. When we plotted the mean mRNA count versus the number of cells each gene is detected in, we observe a large fraction of genes that deviate from the line predicted by our model of technical variation (Fig 2C and 2D). When each gene is colored with our *p*-value statistic, we observe that the more statistically differentially distributed genes tend to have higher mean mRNA values. Interestingly, when we color each gene by the coefficient of variation (standard deviation/mean) we observe the reverse trend: genes with low mean counts have coefficients of variation that are orders of magnitude higher than the more abundantly expressed genes (Fig 2B, 2C and 2D). These statistics indicate that the HVG procedure may be conflating measurement noise with biological variation. Further, if the HVG and DDG methods were to be applied to raw gene counts from the same sample, the two methods may identify non-overlapping feature sets.

### Differentially distributed mRNAs exhibit different modes of variation

**Fig 3:**
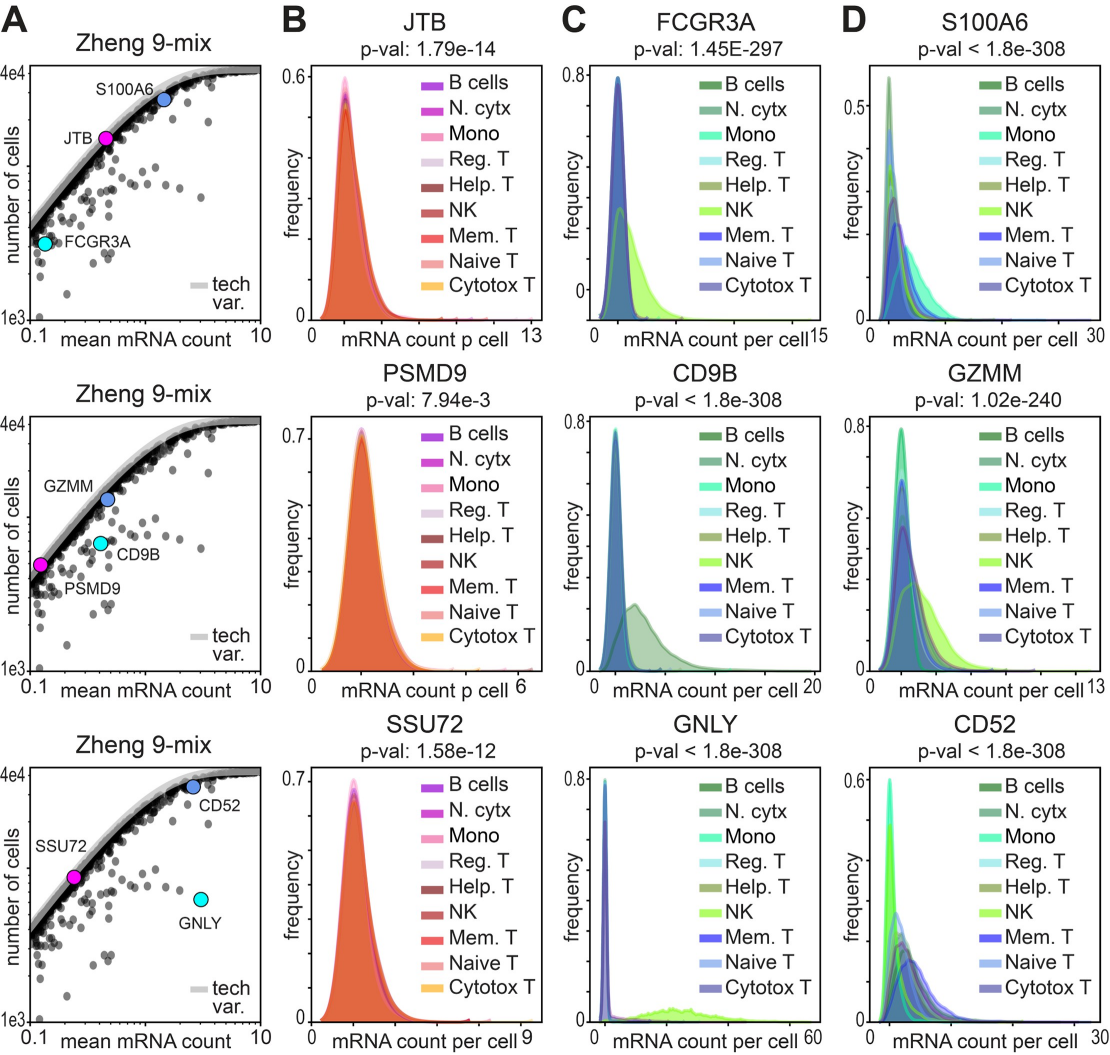
Identification of differentially distributed mRNAs reveals different modes of expression variation. **A)** Scatter of average mRNA count per gene versus the number of cells each gene is identified in for the lymphocyte data, cropped to highlight the three example genes depicted across the row. **B-D)** Kernel density estimation for the distribution of gene counts across cells in the lymphocyte data. The distribution of each gene is plotted for each cell type separately. In each column three example gene distributions are depicted to show B) genes that are not significantly differentially distributed, C) genes that are differentially expressed in specific cell types, and D) genes that quantitatively vary both across and within cell types.

**Fig 4:**
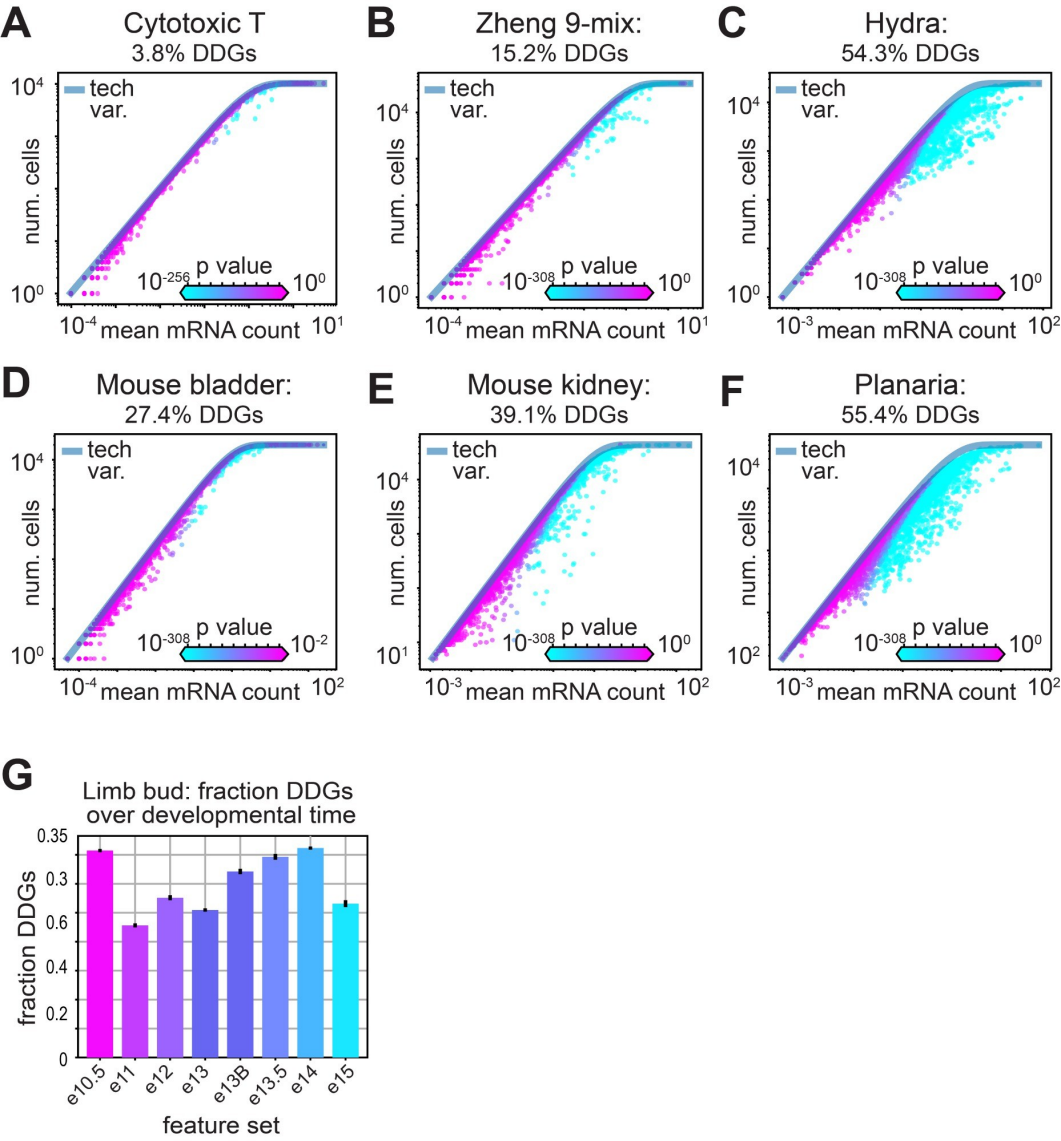
Greater tissue complexity produces a greater fraction of differentially distributed genes. **A-F)** Scatter of average mRNA count per gene versus the number of cells each gene is identified in for the lymphocyte data, where each gene is colored by the computed p-value. The fraction of significantly distributed genes is indicated for A) cytotoxic T cells, B) the Zheng-9 lymphocyte mix, C) hydra, D) mouse bladder, E) mouse kidney, and F) planaria. **G)** Mean fraction of DDGs over developmental time in mouse limb bud data. Error bars show confidence interval around the mean, estimated using the 10 re-sampled sets.

After identifying our sets of DDGs, we next sought to visualize the distribution of these genes across complex tissues. Because cell-identities for the Zheng-9 lymphocyte data have been annotated prior to sampling the transcriptomes, we estimated the probability density for each gene across each of the nine different cell classes. In this orthogonally annotated data, we observed three general patterns of gene expression that can occur independently from mean expression value. 1) For genes with non-significant *p*-values, we find uniform distributions both across and within different cell types (Fig 3B). This class of genes are those genes whose expression levels do not vary more than what would be expected from a binomial capture process. These genes also tend to include, but are not limited to, genes that have mean expression values that are close to zero (Figs 3A and 4). 2) In the Zheng-9 lymphocytes, we observe DDGs that are differentially expressed in specific cell types (Fig 3C). For example, out of the 9 annotated cell types, the gene GNLY is only highly expressed in Natural Killer cells, as it encodes a specialized protein with antimicrobial activity, called granulysin. 3) We also find DDGs that quantitatively vary both across and within cell types (Fig 3D). The proteins encoded by these genes include general cell-function proteins such as ribosomal proteins and S100 family calcium binding proteins, as well as proteins with established immune function. For example, we observed increasing counts of the GZMM serine protease across different classes of activated lymphocytes. We also observed different levels of the CD52 peptide that is associated with the mobility of lymphocytes, as well as other cytokine receptor and antigen associated genes.

### A natural method for determining the number of genes in an scRNA-seq feature set

Several feature selection procedures use an arbitrary cutoff for determining the number of biologically informative genes. In the HVG method, typically 2,000–5,000 of the most variable genes are chosen to create the cell-by-gene distance matrix upon which cell-type clustering is performed. Arbitrary cutoffs are also imposed by the Townes and NBDisp methods, while the M3Drop and NBDrop models use an FDR-adjusted test statistic. Rather than choosing an arbitrary number of genes for an analysis, we hypothesized that we should expect the number of biologically informative genes to increase with the number of different cell identities within a sample. To test this hypothesis, we calculated the fraction of DDGs over samples with increasing tissue complexities.

For tissues with more distributed physiologies, we recovered a greater fraction of differentially distributed genes. For example, we found a fraction of only 3.8% DDGs in an isolated sample of cytotoxic T cells, compared to 15.2% DDGs across the Zheng mix of 9 different lymphocyte cell types (Fig 4A and 4B). Similarly, in the mouse bladder, where three major cell types are expected, 27.4% of the genes are differentially distributed, while we find 39.1% DDGs in the more physiologically diverse mouse kidneys (Fig 4D and 4E). When we calculated the fraction of DDGs across scRNA-seq data collected from whole multicellular organisms, we observed 54.3% DDGs in the hydra and 55.4% DDGs in Planaria (Fig 4C and 4F). We then repeated this experiment using the two other feature selection models where a natural test statistic determines the number of significant genes for each sample. Interestingly, in the NBdrop method, we observed a similar trend as in the DDGs, where the number of genes increased as a function of tissue complexity (S3B Fig). However, the overall number of genes was fewer for each sample, and the kidney sample deviated from the trend with higher numbers of significant genes than the two whole-organism samples. In contrast, the M3drop method produced a fraction of significant genes that decreased with increasing tissue complexity, contradicting our knowledge of gene expression variation across complex tissues (S3C Fig).

We next tested the hypothesis that the fraction of differentially distributed genes increases over developmental time. For this experiment, we used scRNA-seq data collected from mouse limb buds spanning 10.5–15 weeks in embryonic development [40]. To ensure equivalent statistical power, we randomly sampled 5,000 cells from each time point ten times, calculated the fraction of DDGs, and generated confidence intervals across our samples. We found that as developmental time progressed, the fraction of DDGs generally increased, corroborating our hypothesis that diversity of cell types corresponds to diversity in gene expression (Fig 4G). Yet we observed that the earliest and latest limb bud samples (e10.5 and e15) deviated from this trend, potentially due to significant variation in average number of UMI counts per cell detected in these two samples (S3D–S3F Fig). We next sought to investigate how different experimental variables may effect the number of DDGs recovered in a sample.

One critical parameter that often varies between experiment is the total number of UMIs captured in each cell, which is often referred to as “sequencing depth.” To understand how variation in this parameter can alter the number of DDGs that are identified, we performed an experiment where we randomly retained only a fraction of counts from the original Zheng 9 lymphocyte data, as well as data generated from a different data set on cell lines obtained from 10X genomics. To do this, we performed a Bernoulli trial for each UMI count in the data, with a capture probability of 50% or 90%, calculated the set of feature genes, and repeated each experiment 10 times. When we compare the set of DDGs identified using the down-sampled data to the original DDG set, we find that re-sampling the cell line data at either 90% or even 50% probability recovers at least 90% of the original DDGs (S4A Fig). In the Zheng9 case, however, which is already fairly sparse to begin with, there is a larger effect, especially for the 50% probability case (S4C Fig). These results suggest that the set of DDGs identified is robust to minor variation in sequencing depth/coverage, but starts to fail when most of the variation in the data is actually just technical noise; in other words, when there is no signal to detect. Interestingly, when we applied the HVG approach to this experiment, we found no real change in the HVGs, even for the Zheng9 data where the down-sampling produced a dataset with an extreme lack of signal (S4B and S4D Fig). This highlights the fact that the HVG model struggles to separate biological and technical sources of variation.

We next tested the idea that mis-specifying the capture probability parameter, *p*_*c*_, in the DDG model can alter the number of DDGs identified in data. To do this, we titrated the value of *p*_*c*_ from 1% to 80% for a down-sampling experiment similar to that described above, across several datasets used in this study. While we find that the number of DDGs can vary greatly when different *p*_*c*_ values are used, the number of DDGs is relatively stable within the expected experimental range of 5–10% mRNA capture (S5A–S5I Fig).

The fraction of DDGs recovered also depends on how many cells are used to generate the observations. Using the lymphocyte data, where cell types were first identified using FACS prior to scRNA-sequencing, we can approximate a set of “real” DDGs using a supervised approach. We calculated the set of genes whose means are significantly different across the nine cell-type groups, using the Wilcoxon rank-sum test. Next, to evaluate how the number of cells affects the power of our model, we randomly sampled each of the nine types of lymphocytes over a range of 100 to 10,000 cells, then calculated the set of DDGs. As the number of cells increases, we find that the number of DDGs recovered increases linearly (S6A Fig). When we compare the overlap between the predicted DDGs and the “real” DDGs obtained from the Wilcoxon rank-sum test, we find that as we increase the number of cells, the number of “real” DDGs recovered approaches saturation (S6D Fig). Together, these results suggest that the power of the DDG model depends linearly on the number of cells observed, yet the number of biologically variable genes is limited, and with an increasing number of cells, the DDG model can recover an increasing fraction of true variable genes.

### Preservation of variance structure during feature selection

After having validated our DDG model with technical and biological controls, we proceeded to characterize the operational utility of different feature selection methods. In the standard scRNA-seq analysis workflow, feature selection is motivated by the idea that dimensionality reduction can remove axes of variation that arise due to sampling noise, and thus improve the identification of cells that vary in similar and biologically informative ways. Feature selection typically implies a 10-20-fold reduction of genes that are used to represent cells, before the dimensionality is further reduced by principal component analysis, and clustering algorithms are applied. However, the extent to which various feature-selection models can preserve the variance structure of high-dimensional data, or of real biological variation, has yet to be established.

**Fig 5:**
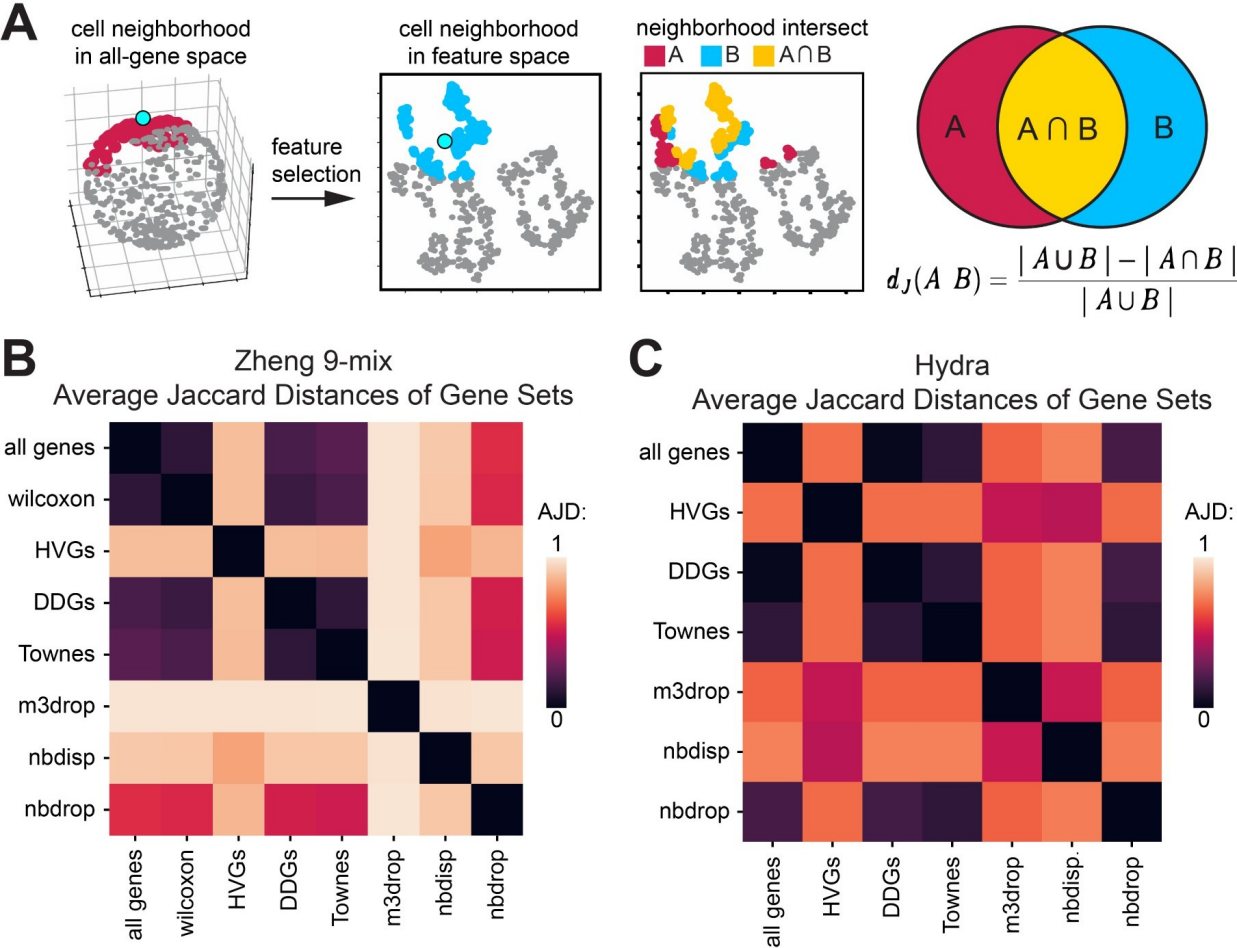
DDGs preserve the structure of variation in high-dimensional scRNA-seq data. **A)** Schematic of the Average Jaccard Distance as a measure of distortion induced by dimensionality reduction **B,C)** Heatmap of pairwise Average Jaccard Distances for each feature set for the B) Zheng-9 lymphocyte mix and C) Hydra.

To evaluate whether cell neighborhood structure can be preserved by various feature selection methods, we calculated the distortion induced to cell neighborhoods by dimensionality reduction using a metric called the Average Jaccard Distance (AJD). The AJD is defined as the per-cell average of the difference between the overlap and total set of k-nearest cell-neighbors in the high-dimensional space, compared to the k-nearest neighbors in the reduced dimensional space (Fig 5A) [41]. If the AJD = 0, all cell neighbors are the same, and no distortion is induced by the dimensionality reduction. In contrast, if the AJD = 1, dimensionality reduction has permuted all of the neighbors, such that none of the original k-nearest neighbors in the high-dimensional data remain in the reduced-dimensional projection.

Using the data where all genes are included as a reference, we calculated the neighborhood distortion induced when various feature selection methods were applied to the Zheng lymphocytes (Fig 5B). Using the set of true biologically varying genes obtained from applying the Wilcoxon test across the FACS sorted lymphocytes, we first established a minimum expectation of distortion induced by feature selection. We found that when cell-neighborhoods were calculated using 20 nearest neighbors and the supervised set of 8,883 genes, the variance structure of the full 20,000 gene set was largely preserved. The Wilcoxon set produced an AJD of only 0.12. In comparison, when the HVGs were used as the basis for the dimensionality-reduced space, the AJD was 0.88 in reference to both the all-gene and Wilcoxon gene neighborhoods. This high level of distortion induced by using just the set of HVGs to construct cell neighborhoods, suggests that the majority of biological variation expressed in the local neighborhood structure has been lost. In contrast, when we calculated the distortion induced by the DDG set, we found a AJD of only 0.19, indicating the DDG set preserves the high-dimensional neighborhood structure at a similar ability of the Wilcoxon genes, even though the DDG set only includes about one third the number of genes as the Wilcoxon set. We next compared the preservation of variance by DDGs to that of other models. We found that the other two binomial-based models more comparable to the preservation of structure by DDGs, with the Townes method producing an AJD of 0.23, and the NBdrop producing greater distortion with an AJD value of 0.56. In contrast the M3drop procedure more significantly permutes the neighborhood structure relative to both the all-gene and Wilcoxon-gene neighborhoods, giving an AJD of 0. 98. These trends were also observed across Hydra–relative to the high-dimensional data, HVGs greatly permutated the neighborhood structures, while DDGs induce almost no distortion (Fig 5C).

### Recovery of biologically meaningful genes by different feature selection methods

**Fig 6:**
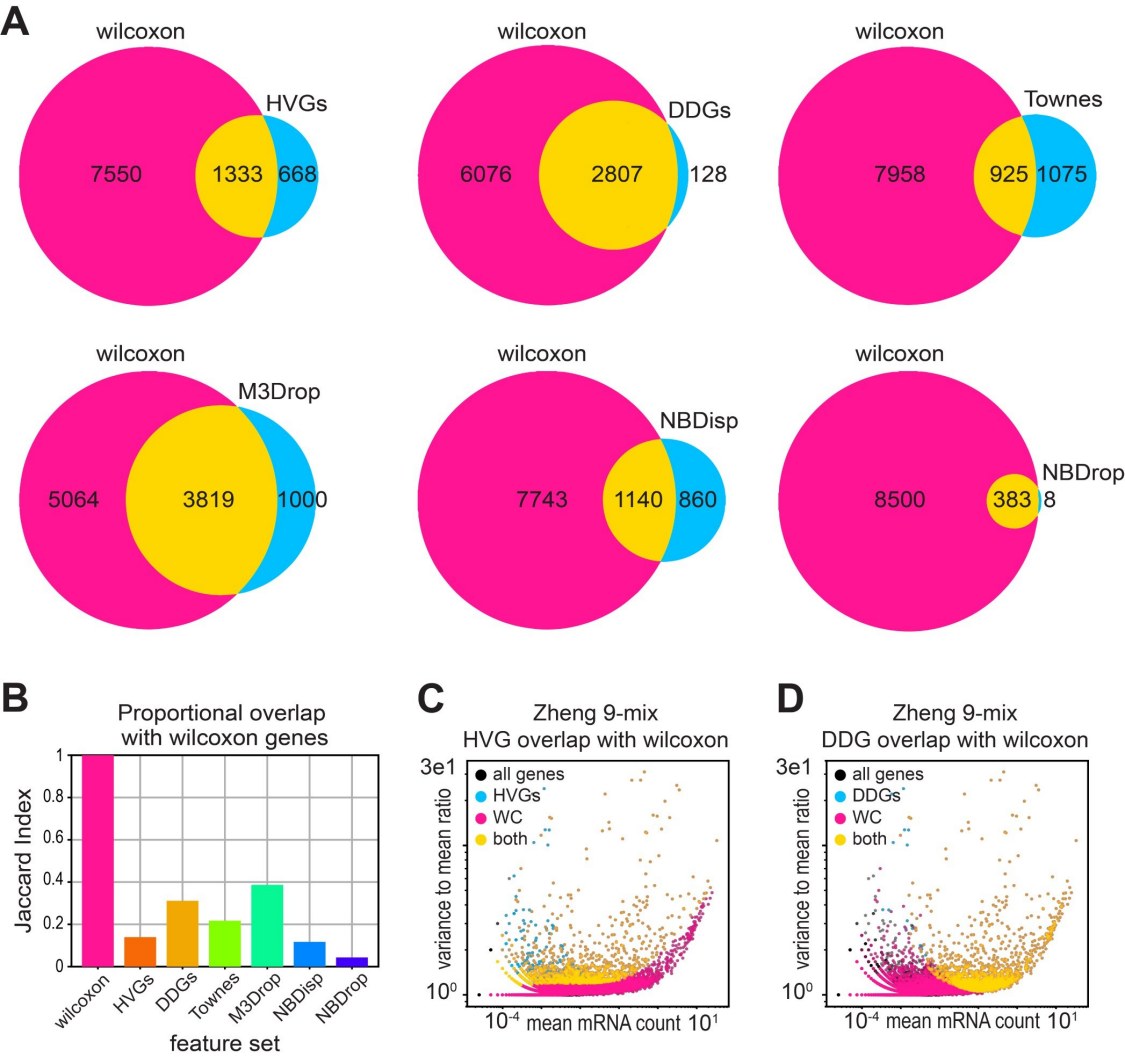
DDGs recover supervised gene-expression differences of FACS-labeled lymphocytes. **A)** Venn diagrams of set overlap for each feature set and the set of genes identified in the Zheng-9 lymphocyte mix using the Wilcoxon rank sum test. **B)** Jaccard index for each feature set illustrating the size-adjusted overlap with the Wilcoxon gene set in the lymphocyte mix. **C,D)** Scatter of the mean mRNA count versus dispersion per gene, colored by set membership for feature sets in the lymphocyte data. C) compares HVGs and Wilcoxon genes while D) compares DDGs and Wilcoxon genes.

We next evaluated whether various feature selection models could recover the specific set of differentially expressed genes that were generated using the supervised Wilcoxon rank-sum test on the FACS-labeled lymphocytes. Out of all the feature selection methods tested, the DDG set recovered the largest fraction of the Wilcoxon genes, specifically sharing 2,807 out of a total of 2,835 DDGs. In contrast, HVGs only recover 1,333 (Fig 6A). We calculated the Jaccard Index to account for the proportional overlap, as different feature selection methods identify different numbers of feature genes in the same data. M3Drop had the highest proportional overlap with the Wilcoxon set, with DDGs having the second highest, while the HVGs had the smallest overlap (Fig 6B).

To further explore this question of capturing biologically meaningful genes, we took a second approach and compared our feature sets with historically established marker genes that are often used in cell-type annotation. We obtained marker gene sets for three datasets including lymphocytes, pancreas, and brain cells from Panglao database [42], and calculated the fraction of marker genes identified by different feature selection models. We find that the M3Drop, followed by the DDG model recover the highest fraction of historically established marker genes (S7B Fig). While Wilcoxon and M3Drop feature sets include a higher number of marker genes compared to the DDGs, the DDG set contains significantly fewer genes, suggesting that a larger proportion of DDGs overlap with historically established cell-type markers (S7A Fig).

Next, to understand these differences in feature sets, and to develop intuition about what kinds of bias may be introduced by various feature selection models, we plotted the mean mRNA count against the “dispersion” (the variance to mean ratio), for each gene in the Zheng lymphocyte data. Here, each gene is colored by its membership to the supervised Wilcoxon set, a particular feature selection set, or membership to both the Wilcoxon and the feature set (Fig 6C and 6D). When we compared the Wilcoxon genes to the HVGs, we observe that HVGs tend to have lower mean expression, but high dispersion (Fig 6C). This finding corroborates the previous hypothesis that the HVG procedure produces a biased output of the most variable genes towards a set of genes with low mean expression but high variance. In contrast, DDGs fail to identify Wilcoxon genes with lower expression levels, potentially due to a lack of power at lower mean expression levels.

We then compared the DDG model to the other more similar, binomial-based methods (S8B–S8D Fig). Interestingly, the genes included in the DDG set are similar to those in Townes, yet each feature set has a unique subset of genes. Additionally, the Townes method tends to select genes that have higher expression means than the DDGs, suggesting a lesser ability to identify differentially expressed genes with lower expression values (S8C Fig). Meanwhile, the NBdrop method produces a set of genes that are a subset of the DDGs, yet have much fewer genes in comparison (S8D Fig). The NBdrop method also tends to select the subset of DDGs that have high dispersion values, again indicating a weaker power in the ability to resolve differentially expressed genes. Taken with the previous findings, these results suggest that compared to other methods, our binomial model with simpler assumptions can identify a greater fraction of true biologically varying genes.

### Recovery of orthogonal labels by different feature sets

We next sought to test the operational goal of feature selection, which is to enable recovery of physiologically similar cells that have similar transcriptional profiles. To evaluate which feature set is most informative for this task, we first used the FACS-labeled lymphocytes, where biological identities are determined *a priori* based on historically-established notions of major immune cell types. In this experiment, we apply the standard approach of Louvain clustering to the dimensionality-reduced data and evaluate how well each feature set can recover the original FACS labels.

For each feature-selected group, we titrated the Louvain resolution parameter from 0.1 to 1, calculated the adjusted rand index (ARI) to compare set membership between Louvain clusters and FACS labels across all cells, and plot the best score per group [43]. In the first experiment, we tested whether clustering feature selected genes using the raw UMI counts, could recover the appropriate lymphocyte cell-types. We found that none of the feature selection groups achieved an ARI score greater than 0.5, indicating a failure to appropriately partition the cells into the historically-established notions of cell-type groups (S9G Fig). Yet, rather than using raw UMI counts, the standard analysis pipeline clusters on feature-selected data that has been CPM and log+1 transformed, and then further reduced by principal component analysis (PCA). The application of PCA is motivated by the idea that reducing the sparseness of the data while retaining the variance structure will enhance the performance of the Louvain clustering algorithm. To test this idea, we performed Louvain clustering at various steps in the analysis pipeline by using raw counts, PCA-transformed counts, and Log+1-CPM-PCA transformed counts. We found that using either PCA-transformed counts or the full log+1-CPM-PCA transformation only marginally improved the performance of various feature selection sets, bringing the best ARI scores to slightly greater than 0.6 (S9H and S9I Fig).

To identify where the Louvain algorithm was failing, we visually inspected the tSNE projections where cells were colored by either their original FACS labels or their Louvain identified clusters [44]. We observed that none of the assayed feature sets were able to recover the appropriate FACS labels for the subsets of related T cell lineages (S9 Fig). This observation corroborates the existing idea that there might not exist a transcriptional basis that can separate these related T cell lineages into distinct groups [44–47]. Based on these observations, we next performed a second FACS-label recovery experiment, where we merged the FACS labels from two subsets of specialized T cell lineages into two distinct supersets. Specifically, we combined naïve cytotoxic cells, cytotoxic T cells, and memory T cells into one set, and naïve T, helper T and regulatory T cells into another set. Using this more granular approach we proceeded to evaluate the performance of different feature sets.

**Fig 7:**
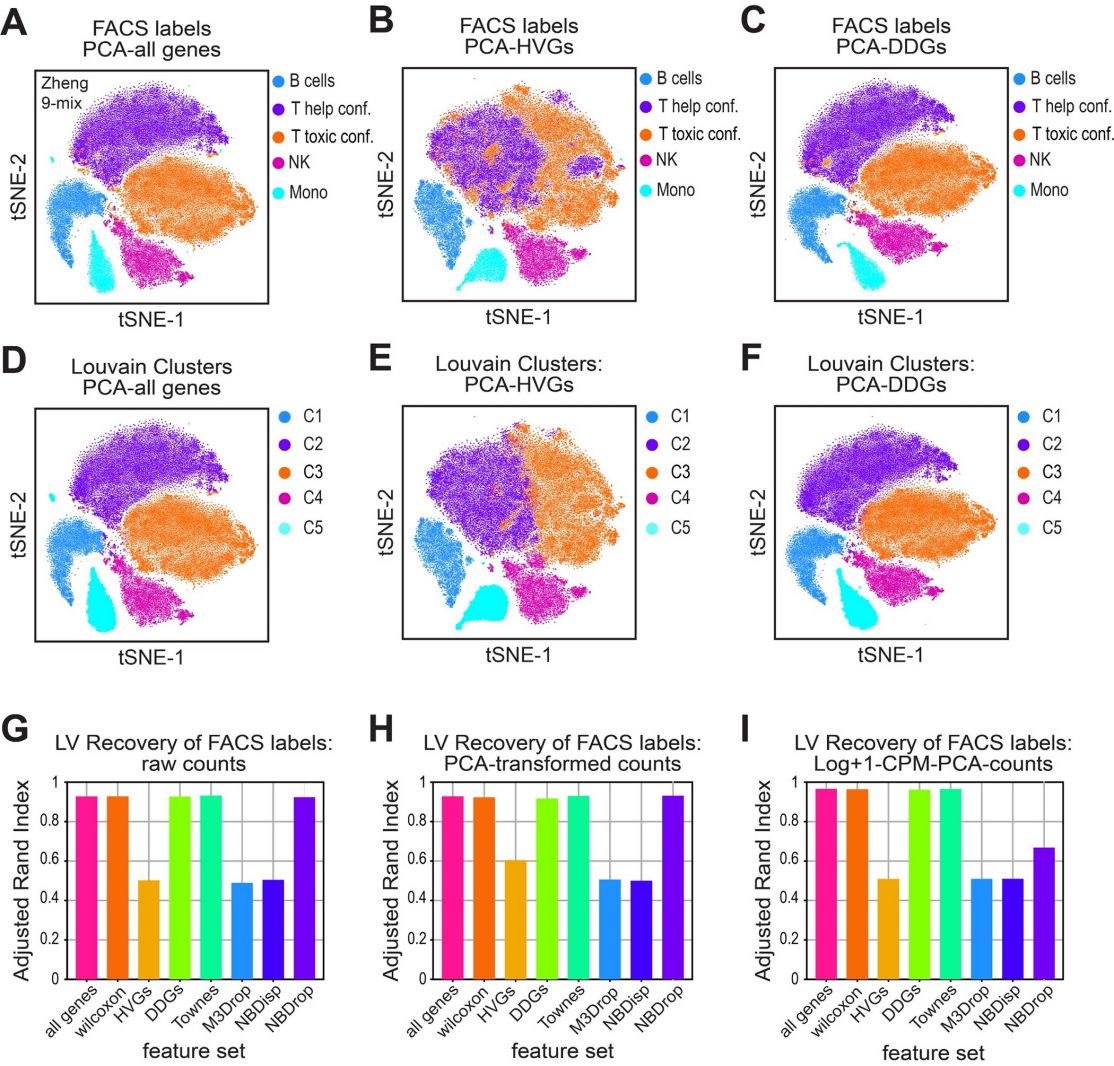
DDGs recover cellular identities of FACS-labeled lymphocytes. **A-F)** tSNE projections for dimensionality-reduced data from the Zheng-9 lymphocyte mix. Principal component analysis was used to reduce (A,D) all genes, (B,E) HVGs, or (C,F) DDGs. Cells are either colored by original FACS-based set membership, where T-cell lineages were merged into two super sets (A-C), or Louvain cluster membership (D-I). **G-I)** Quantification of FACS label recovery by various methods of dimensionality reduction across different feature sets. Louvain clustering was performed across a titration of resolution parameters, cluster labels were compared to original FACS labels where T-cell lineages were merged into two super sets, and the highest adjusted rand index is plotted when G) raw UMI counts, H) PCA-transformed counts, or F) Log+1-CPM-PCA-counts were used as a basis.

Several gene sets were able to recover a large majority of the FACS labels, giving ARI scores over 0.9 (Fig 7). These gene sets include all genes, the Wilcoxon genes, the DDGs and the Townes genes. We found that log+1-CPM-PCA transformed counts again perform slightly better than no transformations or only PCA transformation, potentially by permutating neighborhood structure in a way that is more compatible with the Louvain algorithm (Fig 7G, 7H and 7F). Interestingly, the variance-based approaches, including the HVG set, failed to produce an ARI of greater than 0.6 (Fig 7G, 7H and 7F), even with the minor T-cell lineages merged into major groupings. Inspection of the tSNE projections for the HVG set suggested a loss of the separatrix that distinguishes the two separate T cell lineages. This loss of biologically informative axes of variation in the HVG selected data likely impedes the ability of Louvain algorithm to recover the original FACS labels.

To rule out the possibility that the HVG method was losing biologically relevant information simply due to the choice of too few feature genes, we repeated the label recovery experiment using increasing numbers of HVGs. We first considered cases where the number of HVGs was set to 2,000 (the original number we used, and a typical default value), 4,000 and 8,000. We applied these feature numbers to several datasets with reasonable approaches to orthogonally labeling the “ground truth” of the relevant cell types for clustering: the Zheng 9 FACS-sorted lymphocytes, a multiplexed mixture of B and T cell lines, a multiplexed mixture of A20 and NIH3T3 cell lines, and Citeseq data from spleen and lymph nodes [15,48,49,50]. In the case of the B and T cell lines, and the A20/NIH3T3 cell lines, the cells were labeled before being sequenced in a multiplexed way, allowing for *a priori* identification of the cell line of origin of each cell in the dataset. While the Citeseq data did not do this, it uses oligo-tagged antibodies to measure surface protein expression, allowing us to annotate the cell-type identity of each cell in this dataset in a method analogous to FACS-sorting of historically established marker genes. All the data was subject to the same “standard pipeline” set of transformations (CPM normalization, log transformation, PCA, etc.) before being used as the basis for Louvain clustering.

We found that across all four datasets evaluated, varying the number of HVGs from 2000, 4000, and 8000, had minimal effect on the ability of Louvain clustering to recover the appropriate cell type labels (S10–S14 Fig). In the FACS sorted lymphocytes, regardless of the number of genes used, HVGs only achieve an ARI of about 0.5, indicating a lack of similarity between the FACS labels, and the HVG-based Louvain clusters (S10A–S10C and S12A–S12C Figs). We saw a similar lack of sensitivity to the number of HVGs chosen in the cell line datasets (S11A and S11B Fig), though we should note that all feature selection approaches performed extremely well on these data, likely because cell lines are very transcriptionally distinct and thus are relatively straightforward to cluster effectively. Interestingly, varying the number of HVGs had a slight effect on the ARI for the Citeseq data (S11C Fig). In contrast to the cell line data, however, overall clustering performance on this dataset was quite low (with ARIs around 0.4), which we suspect is due to the fact that the Citeseq protein measurements are less reliable in determining true cell types than the procedure used for FACS.

In analogy to changing the number of HVGs, we also varied the “effective capture probability” parameter in the DDG model, which can strongly influence the number of DDGs identified as significant at any given FDR threshold. For instance, varying the capture probability between 1% and 80% changes the number of significant genes from ~10% to ~75% in the Zheng 9 data (S5B Fig). As with varying the number of HVGs, this has little impact on clustering performance, suggesting that the DDG approach with a reasonably low capture probability (around 5–10%) provides a meaningful basis for clustering (S10D–S10F and S12D–S12G Figs)

At first glance it might seem that the results on both the cell-line and Citeseq data do not provide much additional insight into the utility of various feature selection approaches. As mentioned above, clustering appears to be trivial for the cell-line dataset, as any feature set and any number of features, even randomly selected features, are able to identify the appropriate cell type classification across all cells in the data (S11A and S11B Fig). While this is true for an optimal Louvain cluster resolution parameter, the correct cell-type membership is not typically known *a priori*, and one would not typically be able to determine the optimal clustering resolution without this knowledge. With this in mind, we find that the DDG approach more frequently produces clusters that are concordant with ground truth cell types when a range of cluster resolutions are evaluated (S10 and S12–S14 Figs). In summary, when there are well-defined, known cell identities for each cell in a dataset, the DDG approach performs remarkably well, either outperforming existing feature selection techniques (as in the Zheng9 data) or performing equally well but being far more robust to the choice of parameter values (as in the cell line data).

### Performance of different feature sets in RNA velocity

Finally, as not all scRNA-seq experiments are expected to produce discrete cell type clusters, we tested how the DDG method performed in RNA velocity analysis. RNA velocity uses counts of unspliced and spliced mRNA species to create a model that orders cells along a pseudo-temporal axis, modeling how cells progress through the process of differentiation. Using available data from the pancreas and dentate gyrus, we computed the temporal position of each cell, referred to as the ‘latent time’, using either the HVGs, DDGs, or 2,000 random genes as the input for the RNA velocity package, scVelo [51]. We found that the latent time computed using the DDGs was more similar to the latent time computed using the HVGs, than the results of the DDGs compared to the random genes (S15J–S15M and S16J–S16M Figs). However, since the “ground truth” (i.e. the real velocity for each gene, or the real latent time for each cell) is not known, it is difficult to determine to what extent HVGs or DDGs are capturing the true underlying variation. That being said, it is clear that DDGs provide a useful basis for performing this analysis and do not generate results that are completely out of line with current feature selection approaches. We should note that the surprising performance of the random subset of genes in this experiment suggests that significant further effort is needed in feature selection for RNA velocity analysis.

## Discussion

A major challenge in the study of single cell biology is understanding how variation in gene expression maps to cell physiology. Single-cell RNA sequencing seeks to overcome this challenge by enhancing the resolution at which transcriptional variation can be measured. Yet, the interpretation of these datasets is subject to the precise mapping challenge it seeks to overcome. The dominant approach to interpreting transcriptional variation relies on clustering groups of transcriptionally similar cells, and then comparing differences in gene expression patterns between the groups. Ideally, unsupervised clustering can partition groups of cells that vary in similar ways into biologically meaningful groups. Defining the most appropriate notion of transcriptional similarity and the best way to delineate these cells into cell-type classes remains an ongoing challenge [36,37].

Nonetheless, an operational goal of scRNA-seq studies is to identify genes that are related to biological variation. However, the nature of scRNA-seq data poses challenges for this goal, as the low probability and stochasticity of the scRNA-seq capture process generates sparse, noisy, and extremely high-dimensional mRNA count data. These raw UMI measurements can conflate biological and technical variation and may create challenges for existing unsupervised clustering algorithms. Therefore, a critical step in the analysis lies in developing models of measurement noise that distinguish which axes of variation are biological, and which arise due to technical noise in the measurement.

To achieve this task, the evaluation of null models of biological variation requires empirical support. In this work we propose a feature selection model with minimal assumptions. While our model is similar to the NBDrop and Townes methods, our model relaxes constraints about how cell size affects the mRNA capture process, and treats every mRNA as having an equal probability of being captured. We benchmark how our model performs compared to other popular methods, by using data where known biological variation has been pre-established. Specifically, the Zheng lymphocyte data, where cells had been sorted into major cell type classes and annotated with FACs labels prior to sequencing, enables us to directly map biological variation to transcriptional variation. In addition, because the Zheng lymphocyte data has a relatively large number of cells in the data set, we were able to test model performance using several rigorous metrics.

We demonstrate that, compared to other approaches, our DDG method produces the most accurate mapping of established physiological variation to dimensionality-reduced transcriptional variation. First, the DDG method assigns a *p*-value during the feature selection process, and provides a natural FDR cutoff by which the number of features can be determined after correction for multiple hypothesis testing. We show that, when applied to samples with increasing tissue complexity, this approach is the only method tested that allows us to recover an increasing number of biologically varying genes. Second, DDGs preserve high-dimensional variation in the neighborhood structure of raw and Wilcoxon selected count-by-cell data, suggesting the major axes of transcriptional variation are retained in the reduced dataset. Further, using the orthogonally-annotated lymphocyte data, we find that the DDG feature selection method best recovers a set of supervised differentially expressed genes, and the DDG-set provides a basis for unsupervised clustering that enables recovery of the original FACS cell-identity labels. In other datasets where reasonable ground-truth labels are available, such as data on cell lines, we showed that the DDG approach provides a more robust basis for clustering. DDGs can also be used to identify genes with meaningful biological variation for analyses other than clustering, such as RNA velocity, although more work will be required to fully understand how feature selection influences the results of such analyses.

Taken together, these findings support the use of DDGs in improving the standard scRNA-seq analysis pipeline. Our finding that HVGs severely distort the neighborhood structure of the high-dimensional data and perform poorly across all other tested metrics, corroborates the existing hypothesis that HVGs create an almost arbitrary basis for cell type clustering. We argue that the HVG method biases feature selection toward those genes with low expression values, and that this popular method is not necessarily the most appropriate for dimensionality reduction or feature selection for scRNA-seq data. Moreover, our cell-clustering results suggest feature selection only offers a marginal improvement over clustering cells using the full, raw count by cell matrix. Nonetheless, dimensionality reduction using DDG feature selection can offer an advantage when computational power is limited. DDGs also offer a viable dimensionality reduction approach for other computationally intensive techniques such as manifold learning, when PCA may not be the most appropriate method to identifying manifolds of transcriptional variation at the single cell level.

DDGs identify genes that are differentially distributed in their expression both within a specific subset of specialized cell types, as well as those that vary more continuously both within and across transcriptionally similar groups of cells. Thus, the subset of DDGs provides greater information than standard differential expression tests, which are not designed to identify genes that quantitatively vary across multiple cell classes, or within a particular group. As such, the standard scRNA-seq analysis pipeline overlooks potentially interesting manifolds of transcriptional variation. Overall, this selective vision highlights a more general struggle in biological research. Classifying cells into discrete types can be operationally useful for studying changes in gene expression. However, to what extent transcriptional identity maps to discrete objects, or whether physiologically distinct cell-types correspond to well-separable regions in transcriptional space, remains underexplored [52]. If we cannot reliably draw boundaries around groups of transcriptionally similar cells, the types of questions that we may ask of them are limited. Future work might utilize the DDG method to retain informative axes of biological variation within a more topologically permissive framework for understanding how single-cell variation enables biological organization.

## Methods

### Binomial model of the mRNA capture process

The basis for our null model of variation due to technical noise is quite simple. When run through most scRNA-seq pipelines, cells are lysed in the presence of beads that capture mRNA molecules and then label those mRNAs with both cell-specific barcodes and Unique Molecular Identifiers during the subsequent PCR steps. In our model, we imagine that every mRNA molecule in the cell has the same probability of being captured during this process; we refer to this probability as *p*_*c*_. Say that, for a given gene *i* in cell *j*, there are a total number of *M*_*i*,*j*_ copies of mRNA of that gene in that cell (we use the variable *M* here to denote the “ground truth” number of molecules for that gene in that cell). Under this model, the observed number of mRNAs we obtain in the data will follow a simple binomial distribution:

$$p(m_{i,j}|M_{i,j})=\left(\begin{array}{c} M_{i,j}\\ m_{i,j} \end{array}\right)p_{c}^{m_{i,j}}{\left(1-p_{c}\right)}^{M_{i,j}-m_{i,j}}$$

where *m*_*i*,*j*_ is just the observed number of UMI counts for the mRNA for gene *i* in cell *j*.

### Expected number of cells expressing a gene *i*

A critical parameter in this model is obviously the ground truth amount of mRNA in each cell for each gene, *M*_*i*,*j*_. We obviously don’t know this number, since all we have are the observed values of *m*_*i*,*j*_. The simplest null model for *M*_*i*,*j*_ would be that there is actually *no biological variation* in the mRNA levels for gene *i* in the population. In other words, imagine that we had a situation where *every single cell* started out with the same number of copies of mRNA for that gene (i.e. *M*_*i*,*j*_ = *M*_*i*,*k*_ ∀*j*, *k*). Of course, even in this scenario, there would be some variation in the resulting scRNA-seq data, since the sampling process is binomial and there would be some differences between cells simply because the capture process is stochastic. Our ultimate goal is to identify genes whose expression pattern is inconsistent with this null expectation; in other words, genes whose variation within the data *cannot be explained* purely on the basis of the stochastic process of mRNA capture during the experiment, i.e. technical noise. For simplicity, we call this constant amount of mRNA in each cell in this simple model *M*_*i*_, since the amount of mRNA no longer depends on the identity of the cell.

In this model, it is natural to consider the relationship between the *number of cells* that express a gene *i* (i.e. the number of cells with *m*_*i*,*j*_>0, call this *N*_*c*,*i*_) and the average expression of that gene in the population *E*(*m*_*i*_) (note here that we drop the index *j* since we have averaged over all the cells). The reason for doing this is the fact that the binomial model above immediately suggests a simple relationship between these two observations. To understand that relationship, we first have to estimate the value of *M*_*i*_, the constant amount of mRNA that each cell had to start with in this model. If we assumed our model was true, and subjected all the cells to this binomial sampling process, this would give us *E*(*m*_*i*_) = *p*_*c*_*M*_*i*_; in other words, the average amount of mRNA actually observed in this experiment would be the capture probability times the number of mRNA molecules we started with. Inverting this equation gives us an estimate of *M*_*i*_:

$$M_{i}=\frac{E(m_{i})}{p_{c}}$$

which is the maximum-likelihood estimator of this parameter under the null model. Note that we can easily calculate *E*(*m*_*i*_) empirically for any gene in our dataset. As discussed in the main text, we don’t actually know the capture probability *p*_*c*_ for any given dataset, so this becomes essentially the only external parameter we set in this model. Our findings suggests that the results of the DDG model are generally insensitive to the specific value chosen for *p*_*c*_, at least for most datasets. Experimental estimates suggest that *p*_*c*_ is in the range of 5–10% [5], so we suggest choosing a value in that range for most cases. We set this value to 5% for the bulk of our own analyses, unless otherwise specified.

The question we now want to address is: on average, how many cells in our dataset will express any given gene *i* under this simple null model? Call the total number of cells within the dataset *N*_*T*_, and recall that the number of cells that express this particular gene is *N*_*c*,*i*_. To calculate this expectation, we first realize that the probability of having *exactly* 0 mRNA molecules detected for gene *i* is ${\left(1-p_{c}\right)}^{M_{i}}$, which is just the probability of failing to capture a single mRNA molecule despite having *M*_*i*_ trials in which to do so. Of course, we have our estimate *M*_*i*_ = *E*(*m*_*i*_)/*p*_*c*_ above, which allows us to write this probability in terms of the observed average for the expression level of the gene and the capture probability parameter. Now, note that, if ${\left(1-p_{c}\right)}^{M_{i}}$ is the probability of having exactly 0 mRNA molecules for this gene, then $1-{\left(1-p_{c}\right)}^{M_{i}}$ is the probability of having *at least 1* copy of the mRNA in any given cell. This immediately leads to the following expression:

$$E(N_{c,i})=N_{T}\left(1-{\left(1-p_{c}\right)}^{\frac{E(m_{i})}{p_{c}}}\right),$$

which relates the expected value of the number of cells expressing the gene, *N*_*c*,*i*_, to the observed average mRNA level for the gene *E*(*m*_*i*_) using just one parameter *p*_*c*_. This results in the characteristic curve shown in Figs 2, 3 and 4.

### p-value calculation under the null model

Real datasets obviously contain genes where the number of cells that express that gene in the dataset is lower than the expected value based on this simple null model (see, e.g., the many points that lie below the curve in Fig 2C). It is unclear, however, to what extent any variation from this line represents statistically significant deviation from that expectation. To quantify that, we can calculate a p-value for observing a certain number of cells expressing any given gene, given its observed average expression level *E*(*m*_*i*_). To do that, first define the *observed* number of cells expressing this gene *i* as *N*_*c*,*i*_. Also, as discussed above, we have the probability that any given cell will *not* express the gene. For simplicity, we re-define this probability as *p*_0_(*i*), the probability of having 0 counts for gene *i*:

$$p_{0}(i)\equiv {\left(1-p_{c}\right)}^{\frac{E(m_{i})}{p_{c}}}.$$

Now, notice that, under our null model, we can actually write down the probability of observing a certain value of *N*_*c*,*i*_. This is actually just another Binomial sampling process: for every cell, it can either express the gene or not. This gives us a very simple expression for the probability of observing a certain number of cells expressing this gene:

$$p(N_{c,i})=\left(\begin{array}{c} N_{T}\\ N_{c,i} \end{array}\right){\left(1-p_{0}(i)\right)}^{N_{c,i}}{p_{0}(i)}^{N_{T}-N_{c,i}}.$$

which obviously gives the equation in the section above as its average. To obtain a p-value, all we do is sum this probability over all possible values of the observed number of cells expressing the gene, from 0 to the observed number:

$$p‐\mathrm{value}=\sum_{N=0}^{N_{c,i}}p(N).$$

Since there are a large number of genes in any given dataset (~20,000 or so), application of this model to determine a group of “significant” DDGs requires correction for multiple hypothesis testing. As is standard in these types of approaches, we apply the Benjamani-Hochberg approach to control the familywise error in the calculation. As such, one can specify the false discovery rate that one is willing to tolerate: based on our results for the ERCC controls, we ourselves set this to be 1% for the calculations presented here, but one can choose this number to be more or less stringent depending on the circumstances.

Of course, this null model is extremely simplistic, as it posits absolutely no underlying variation in the “true” amount of mRNA in each cell, *M*_*i*,*j*_, and is thus a model for cases where variation in the observed mRNA levels can be explained by a model in which that variation is *purely* technical. Interestingly, our results reveal that, despite the simplicity of the model, many datasets have a large number of genes whose variation is indistinguishable from this null model. This includes a large number of HVGs, which is perhaps not surprising given the low expression levels observed in these gene sets. Nonetheless, it is important to note that many such genes have gene expression patterns that cannot be statistically distinguished from purely technical variation. One could, of course, relax the constraint that *M*_*i*,*j*_ is constant for all cells. This would be equivalent to coming up with some null distribution of *M*_*i*,*j*_ values where the variation in these starting values would be real, but not biologically interesting. Doing so, however, requires making strong assumptions about what such uninteresting variation might look like. As such, here we focus on this simple null model that distinguishes cases where all the observed variation could explained purely through technical noise. We leave exploration of alternative models to future work.

All code for calculation of DDG p-values may be found at:

https://github.com/DeedsLab/Differentially-Distributed-Genes

### Gaussian Mixture Model

To test the DDG model described above, we generated a simple GMM that allows us to specify *a priori* a set of genes that have meaningful biological variation and a set of genes that do not. The idea here was to sample a set of values of *M*_*i*,*j*_ for a group of cells from a Gaussian, simulating a distribution of “ground truth” mRNA values, and then apply the binomial capture process to these mRNA levels to obtain simulated “observed” *m*_*i*,*j*_ values. To do this, we created a simulated dataset with 3 distinct cell types and 1000 total genes. For each cell type, we had 300 “marker genes” for that cell type. A marker gene has two states: cells in which it is “high” and cells in which it is “low;” the idea here is that the marker gene is high in the cells that are of the right type, and low in all the other cells. In other words, say we call our three cell types A, B, and C. For cell type A, there would be 300 genes where every cell in cell type A has high expression for that gene, and every cell from cell types B and C would have low expression of that gene. Since there are 300 genes for each cell type, this leaves us with 100 “ubiquitously” expressed genes, which we took to be low in all cells.

To generate this simulated data, we had a different Gaussian for these two states, high and low. To estimate parameters for these Gaussians, we used the data from the A20 and 3T3 cell lines discussed above, since these are very clearly different cells and have many differentially expressed marker genes. We analyzed count distributions of genes that were both relatively highly expressed (had observed averages expression levels of about 1 and were significantly differentially expressed between the two cell types according to the standard Wilcoxon Rank-Sum test). Looking at these data, we chose to set the average to 35 and standard deviation to 2 for genes in the “high” expression state. We used an average of 15 and a standard deviation of 1 for genes in the low expression state. Note that, since counts are inherently discrete and this GMM produces non-integer values, we used the standard round() function to round the sampled numbers to the nearest integer value.

To generate the simulated dataset, we independently sampled values for all 1000 genes in cells of type A, B, and C. We simulated 3,000 cells of each type for a total of 9,000 cells. After generating a gene expression vector for each cell in this model, we simulated the experiment by independently executing the binomial capture model described above for each gene in each cell. In other words, the simulation provided us with an *M*_*i*,*j*_ value for each gene in each cell, and we used that to sample observed *m*_*i*,*j*_ values using the binomial sampling process described above. We used a capture probability of 5% for this particular simulation. The resulting simulated data was then subjected to the DDG analysis pipeline described above.

## Supporting information

S1 Fig **A-C)** Kernel density estimation for the distribution of average gene expression across cells for each gene cluster group in A) mouse bladder, B) mouse kidney, and C) Planaria. **D-F)** Kernel density estimation for the distribution of fraction of cells each gene in the gene cluster is identified in D) mouse bladder, E) mouse kidney, and F) Planaria.
(TIF)

S2 Fig A simulation scheme to test ground-truth marker gene recovery by DDG and HVG models. A gaussian mixture model (GMM) with 3 cell types, 3000 cells per cell type, and 1000 genes per cell type was generated. For each cell-type, 300 genes were chosen as ‘marker genes’ and the count values were drawn from a Gaussian with a mean of 35 and standard deviation of 2. If a gene is not a marker gene, the count value was drawn from a gaussian with a mean of 15 and standard deviation of 1. As a result, there are a total of 900 ground truth marker genes, with 300 in each cell type. The model was then down-sampled by performing a Bernouli trial for each gene count in the data, varying the “capture probability’ from 1% to 50%. **A)** Fraction of counts with a value of 0 in each down-sampled trial. **B)** Fraction of genes identified as DDGs when our DDG model was applied to each down-sampled version of the GMM. Here, the capture probability was set to the exact value used in the simulated experiment. **C)** Fraction of ground truth marker genes contained in the DDG set, when the DDG model used the accurate capture probability. **D)** Fraction of ground truth marker genes contained in the HVG set when the HVG model was specified to recover 900 HVGs for each down-sampled trial. **E)** Fraction of genes identified as DDGs when the DDG model parameter for capture probability was set at 5%. **F)** Fraction of ground truth marker genes contained in the DDG set, when the DDG model parameter for capture probability was set at 5%.
(TIF)

S3 Fig **A-C)** Mean fraction of significant features over increasing tissue complexity using A) the DDG method, B) the NBDrop method, and C) the M3Drop method. **D-F)** Quality control metrics for the limb bud data. A) Mean total number of genes for each sample, with error bars depicting confidence intervals around the mean. B) Mean total number of UMI counts for each sample, with error bars depicting confidence intervals around the mean. C) Distribution of UMI counts per cell in each limb bud sample.
(TIF)

S4 Fig Modeling the effect of “sequencing depth” on feature selection. A20 & NIH3T3 cell line data (A,B) and Zheng 9 lymphocyte data (C,D) was down-sampled by performing a Bernoulli trial for each UMI count in the data, using a 50% or 90% rate. Each experiment was repeated 10 times, and standard deviation bars are plotted on each graph. **A)** Fraction of the original A20 & NIH3T3 DDG set that was recovered in the down-sampled cases. **B)** Fraction of the original A20 & NIH3T3 HVG set that was recovered in the down-sampled cases. **C)** Fraction of the original Zheng 9 DDG set that was recovered in the down-sampled cases. **D)** Fraction of the original Zheng 9 HVG set that was recovered in the down-sampled cases.
(TIF)

S5 Fig **The effect of the capture probability parameter on DDG selection A-I)** For each dataset, the capture probability parameter in the DDG model, *p*_*c*_, was titrated from 1% up to 80%. Bar graphs illustrate the number of DDGs identified at different *p*_*c*_, values for A) 10x scRNA-seq data generated from Cytotoxic T cells purified by FACs, B) 10x scRNA-seq data generated from the full set of Zheng 9 lymphocytes, C) Citeseq data generated from Spleen and Lymph cells, D) 10x scRNA-seq data generated from the A20 cell line, E) 10x scRNA-seq data generated from the NIH3T3 cell line, F) 10x scRNA-seq data generated from A20 and NIH3T3 cell lines, G) 10x scRNA-seq data generated from the Raji B cell line, H) 10x scRNA-seq data generated from the Jurkat T cell line, and I) 10x scRNA-seq data generated from the Raji B and the Jurkat T cell lines.
(TIF)

S6 Fig Modeling the effect of number of cells on DDG selection. **A-D)** Empirical power estimations for the DDG method using the Zheng-9 lymphocyte mix, where a new set of DDGs was computed with increasing sample size. A) Mean number of DDGs as a function of increasing number of cells per cell type in the Zheng-9 lymphocyte mix. B) Mean total number of genes as a function of increasing cell number per sample. C) Mean overlap of new DDG sets with original DDG set calculated from the full, 5k cells per type Zheng-9 lymphocyte data. D) Mean overlap of new DDG sets with original Wilcoxon set of differentially expressed genes, as a function of number of cells per cell type.
(TIF)

S7 Fig Comparison of feature sets with historically established marker genes. Cell type specific marker gene lists were downloaded from the Panglao database and used for this analysis. **A)** Number of genes in each feature set for the Zheng 9 lymphocytes. **B)** Fraction of marker gene set that is recovered in each feature set of the Zheng 9 lymphocytes. **C)** Quantification of FACS label recovery when the full set of marker genes were added to each feature set or when each feature set was used alone. Louvain clustering was performed across a titration of resolution parameters, cluster labels were compared to original FACS labels where T-cell lineages were merged into two super sets, and the highest adjusted rand index is plotted when log+1-CPM-PCA-counts were used as a basis. **D)** Fraction of marker gene set that is recovered in each feature set of the pancreas data. **E)** Fraction of marker gene set that is recovered in each feature set of the dentate gyrus data.
(TIF)

S8 Fig **A)** Venn diagram of set overlap for all feature sets calculated for the Zheng-9 lymphocyte mix. **B)** Venn diagrams of set overlap for the DDGs and the two other binomial based feature selection methods applied to the lymphocyte data. **C,D)** Scatter of the mean mRNA count versus dispersion per gene, colored by set membership for feature sets in the lymphocyte data. C) compares the Townes genes and DDGs while D) compares the NBDrop genes and DDGs.
(TIF)

S9 Fig **A-F)** tSNE projections for dimensionality-reduced data from the Zheng-9 lymphocyte mix. Principal component analysis was used to reduce (A,D) all genes, (B,E) HVGs, or (C,F) DDGs after log+1 and CPM transforming the count data. Cells are either colored by original FACs-based set where T-cell lineages were merged into two super sets (A-C) or Louvain cluster membership (D-F). **G-I)** Quantification of FACS label recovery by various methods of dimensionality reduction across different feature sets. Louvain clustering was performed across a titration of resolution parameters, cluster labels were compared to original FACS labels where T-cell lineages were merged into two super sets, and the highest adjusted rand index is plotted when G) raw UMI counts, H) PCA-transformed counts, or F) log+1-CPM-PCA-counts were used as a basis.
(TIF)

S10 Fig **A-F)** Quantification of FACS label recovery by various methods of dimensionality reduction across different feature sets. Louvain clustering was performed across a range of resolution parameters, cluster labels were compared to original FACS labels where T-cell lineages were merged into two supersets, and the highest adjusted rand index is plotted when A) raw UMI counts of HVGs, B) PCA-transformed HVG counts, C) log+1-CPM-PCA-HVG counts, D) raw UMI counts of DDGs, B) PCA-transformed DDG counts, C) log+1-CPM-PCA-DDG counts were used as a basis.
(TIF)

S11 Fig **A-C)** Quantification of orthogonal label recovery by various methods of dimensionality reduction across different feature sets. Louvain clustering was performed across a titration of resolution parameters, cluster labels were compared to cell-type labels, and the highest adjusted rand index is plotted when A) log+1-CPM-PCA counts from the multiplexed A20 and NIH3T3 cell lines, B) log+1-CPM-PCA counts from the multiplexed B and T cell lines, or C) log+1-CPM-PCA counts from the cell-surface protein gated Citeseq data, were used as a basis.
(TIF)

S12 Fig **A-G)** Quantification of FACS label recovery by various methods of dimensionality reduction across different feature sets and different Louvain clustering resolution parameters for the Zheng 9 lymphocyte data. Louvain clustering was performed across a titration of resolution parameters, cluster labels were compared to original FACS labels where T-cell lineages were merged into two super sets, and the adjusted rand index is plotted when A) log+1-CPM-PCA counts of 2e3 HVGs, B) 4e3 HVGs, or C) 8e3 HVGs, or log+1-CPM-PCA counts of DDGs identified with a *p*_*c*_ of D) 1%, E) 2%, F) 5%, or G) 10% were used as a basis.
(TIF)

S13 Fig **A-H)** Quantification of multiplex label recovery by various methods of dimensionality reduction across different feature sets and different Louvain clustering resolution parameters for the multiplexed A20 and NIH3T3 cell line data. Louvain clustering was performed across a titration of resolution parameters, cluster labels were compared to original cell-type labels and the adjusted rand index is plotted when A) Log+1-CPM-PCA counts of all genes, B) log+1-CPM-PCA counts of 2e3 HVGs, C) 4e3 HVGs, or D) 8e3 HVGs, or log+1-CPM-PCA counts of DDGs identified with a *p*_*c*_ of E) 1%, F) 2%, G) 5%, or H) 10% were used as a basis.
(TIF)

S14 Fig **A-H)** Quantification of multiplex label recovery by various methods of dimensionality reduction across different feature sets and different Louvain clustering resolution parameters for the multiplexed B and T cell line data. Louvain clustering was performed across a titration of resolution parameters, cluster labels were compared to original cell-type labels and the adjusted rand index is plotted when A) Log+1-CPM-PCA counts of all genes, B) log+1-CPM-PCA counts of 2e3 HVGs, C) 4e3 HVGs, or D) 8e3 HVGs, or log+1-CPM-PCA counts of DDGs identified with a *p*_*c*_ of E) 1%, F) 2%, G) 5%, or H) 10% were used as a basis.
(TIF)

S15 Fig RNA velocity analysis of Pancreas data using different feature sets. **A-C)** RNA velocity was performed using the scVelo software package and HVGs as the basis. A) UMAP projection of pancreas cells colored by HVG-based velocity and cluster membership. B) UMAP projection of pancreas cells colored by HVG-inferred latent time. C) Heatmap of gene counts of the 300 top genes contributing to the HVG-based latent time estimate, where each row represents a gene and each column represents individual cell that is ordered along the latent time axis. **D-F)** RNA velocity was performed using the scVelo software package and DDGs as the basis. D) UMAP projection of pancreas cells colored by DDG-based velocity and cluster membership. E) UMAP projection of pancreas cells colored by DDG-inferred latent time. F) Heatmap of gene counts of the 300 top genes contributing to the DDG-based latent time estimate, where each row represents a gene and each column represents individual cell that is ordered along the latent time axis. **G-I)** RNA velocity was performed using the scVelo software package and 2e3 random genes as the basis. G) UMAP projection of pancreas cells colored by random gene-based velocity and cluster membership. H) UMAP projection of pancreas cells colored by random gene-inferred latent time. I) Heatmap of gene counts of the 300 top genes contributing to the random gene-based latent time estimate, where each row represents a gene and each column represents an individual cell that is ordered along the latent time axis. **J-L)** Scatter plots of latent times assigned to each cell for J) HVGs and DDGs, K) HVGs and random genes, and L) DDGs and random genes. **M)** Pearson correlations of latent times inferred for each feature set compared.
(TIF)

S16 Fig RNA velocity analysis of Dentate Gyrus data using different feature sets. **A-C)** RNA velocity was performed using the scVelo software package and HVGs as the basis. A) UMAP projection of dentate gyrus cells colored by HVG-based velocity and cluster membership. B) UMAP projection of dentate gyrus cells colored by HVG-inferred latent time. C) Heatmap of gene counts of the 300 top genes contributing to the HVG-based latent time estimate, where each row represents a gene and each column represents individual cell that is ordered along the latent time axis. **D-F)** RNA velocity was performed using the scVelo software package and DDGs as the basis. D) UMAP projection of dentate gyrus cells colored by DDG-based velocity and cluster membership. E) UMAP projection of dentate gyrus cells colored by DDG-inferred latent time. F) Heatmap of gene counts of the 300 top genes contributing to the DDG-based latent time estimate, where each row represents a gene and each column represents individual cell that is ordered along the latent time axis. **G-I)** RNA velocity was performed using the scVelo software package and 2e3 random genes as the basis. G) UMAP projection of dentate gyrus cells colored by random gene-based velocity and cluster membership. H) UMAP projection of dentate gyrus cells colored by random gene-inferred latent time. I) Heatmap of gene counts of the 300 top genes contributing to the random gene-based latent time estimate, where each row represents a gene and each column represents an individual cell that is ordered along the latent time axis. **J-L)** Scatter plots of latent times assigned to each cell for J) HVGs and DDGs, K) HVGs and random genes, and L) DDGs and random genes. **M)** Pearson correlations of latent times inferred for each feature set compared.
(TIF)
