# Supplementary figures and images for: Binomial models uncover biological variation during feature selection of droplet-based single-cell RNA sequencing
Source: PLoS Comput Biol. 2024 Sep 6;20(9):e1012386. doi: 10.1371/journal.pcbi.1012386 (PMC11410258; doi:10.1371/journal.pcbi.1012386)

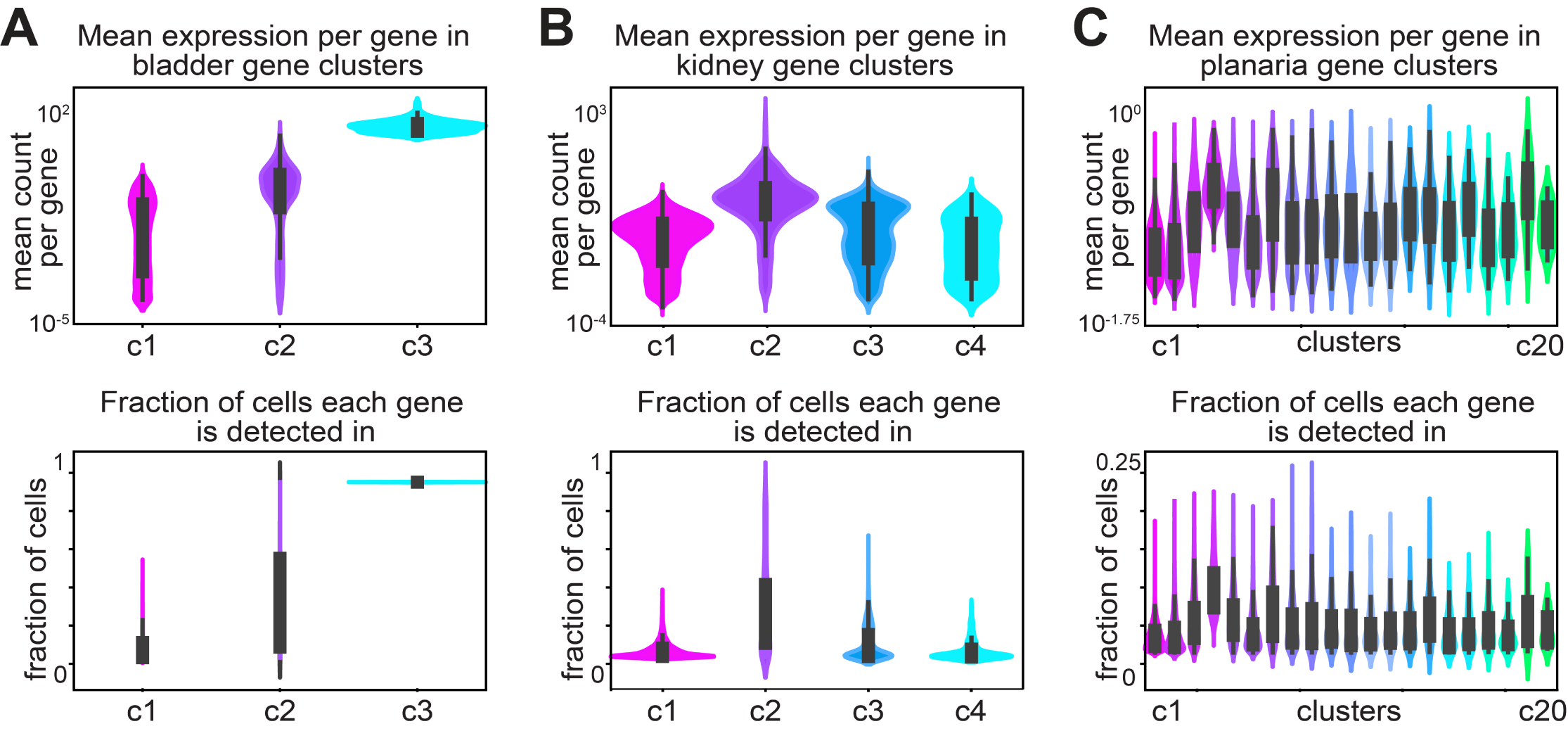

Supplement: S1 Fig — A-C) Kernel density estimation for the distribution of average gene expression across cells for each gene cluster group in A) mouse bladder, B) mouse kidney, and C) Planaria. D-F) Kernel density estimation for the distribution of fraction of cells each gene in the gene cluster is identified in D) mouse bladder, E) mouse kidney, and F) Planaria. (TIF) [file pcbi.1012386.s001.tif]

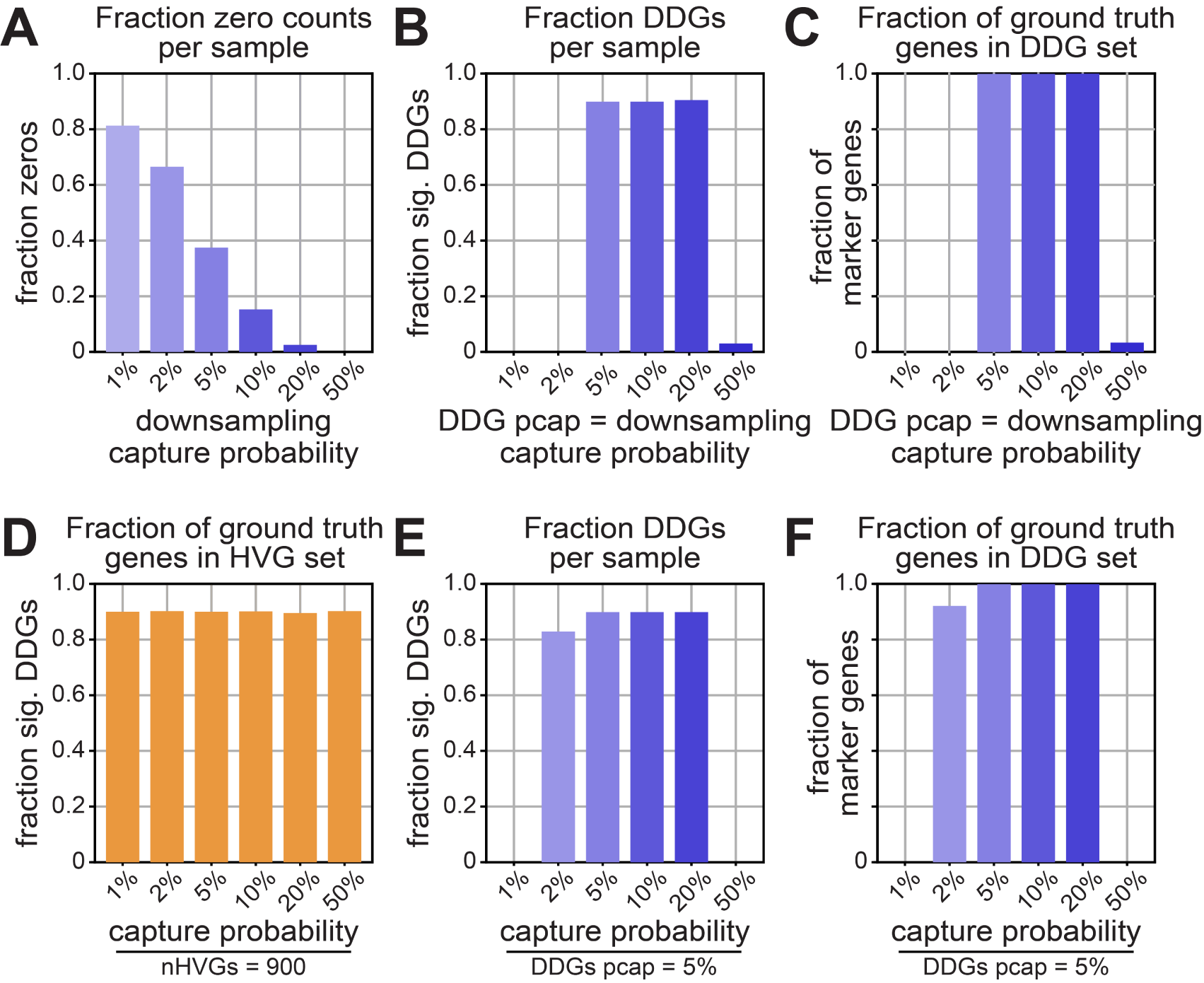

Supplement: S2 Fig — A gaussian mixture model (GMM) with 3 cell types, 3000 cells per cell type, and 1000 genes per cell type was generated. For each cell-type, 300 genes were chosen as ‘marker genes’ and the count values were drawn from a Gaussian with a mean of 35 and standard deviation of 2. If a gene is not a marker gene, the count value was drawn from a gaussian with a mean of 15 and standard deviation of 1. As a result, there are a total of 900 ground truth marker genes, with 300 in each cell type. The model was then down-sampled by performing a Bernouli trial for each gene count in the data, varying the “capture probability’ from 1% to 50%. A) Fraction of counts with a value of 0 in each down-sampled trial. B) Fraction of genes identified as DDGs when our DDG model was applied to each down-sampled version of the GMM. Here, the capture probability was set to the exact value used in the simulated experiment. C) Fraction of ground truth marker genes contained in the DDG set, when the DDG model used the accurate capture probability. D) Fraction of ground truth marker genes contained in the HVG set when the HVG model was specified to recover 900 HVGs for each down-sampled trial. E) Fraction of genes identified as DDGs when the DDG model parameter for capture probability was set at 5%. F) Fraction of ground truth marker genes contained in the DDG set, when the DDG model parameter for capture probability was set at 5%. (TIF) [file pcbi.1012386.s002.tif]

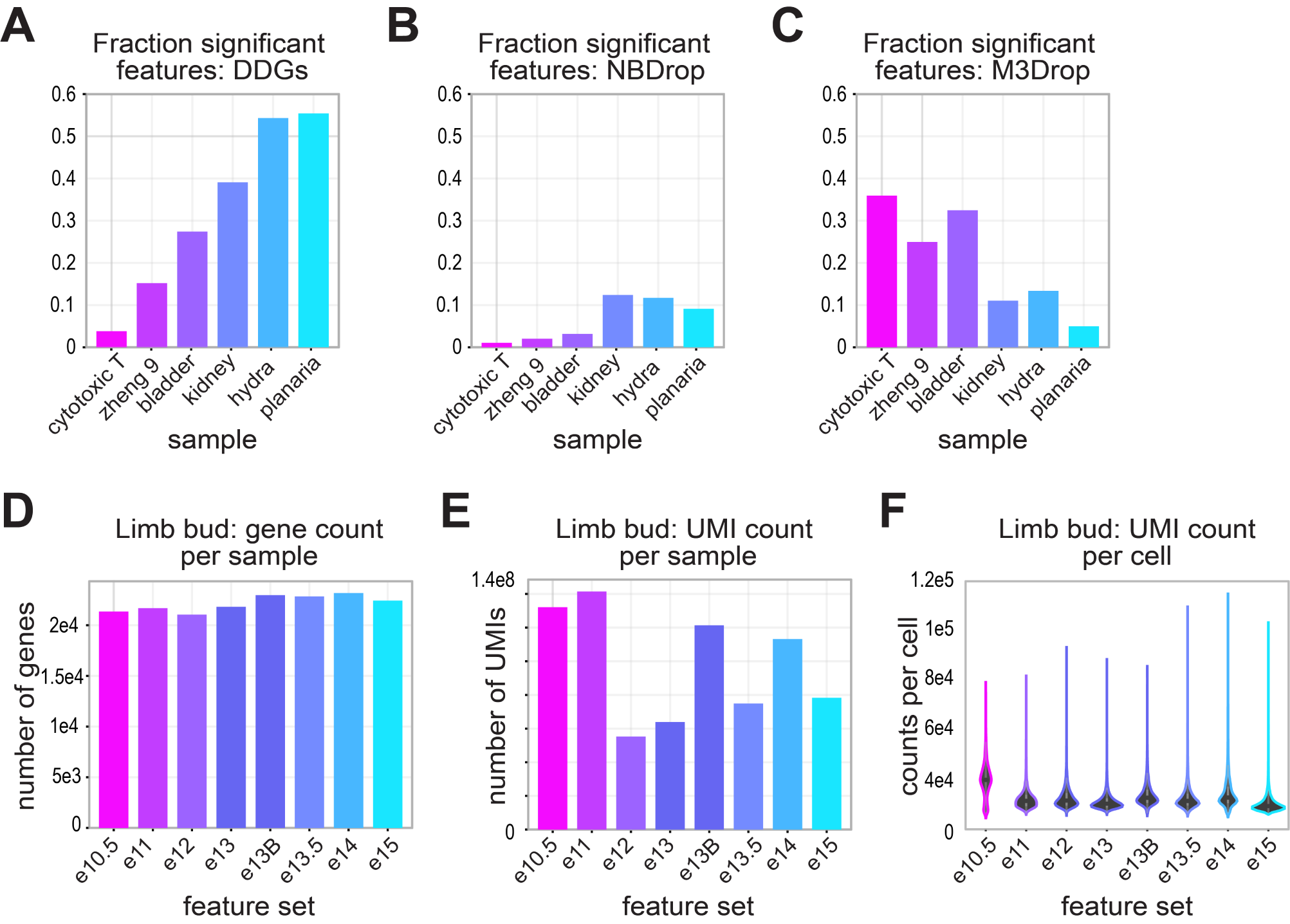

Supplement: S3 Fig — A-C) Mean fraction of significant features over increasing tissue complexity using A) the DDG method, B) the NBDrop method, and C) the M3Drop method. D-F) Quality control metrics for the limb bud data. A) Mean total number of genes for each sample, with error bars depicting confidence intervals around the mean. B) Mean total number of UMI counts for each sample, with error bars depicting confidence intervals around the mean. C) Distribution of UMI counts per cell in each limb bud sample. (TIF) [file pcbi.1012386.s003.tif]

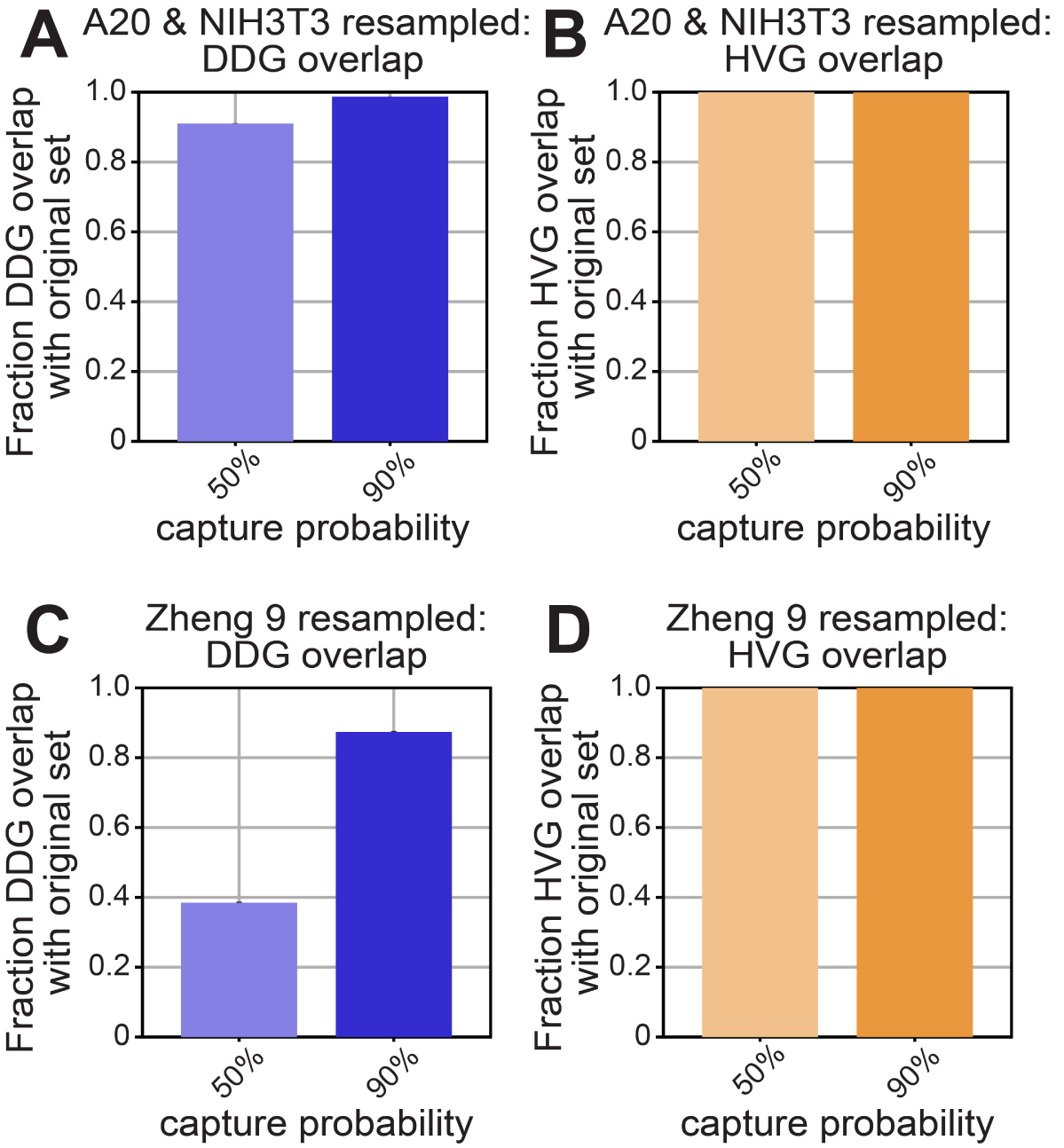

Supplement: S4 Fig — A20 & NIH3T3 cell line data (A,B) and Zheng 9 lymphocyte data (C,D) was down-sampled by performing a Bernoulli trial for each UMI count in the data, using a 50% or 90% rate. Each experiment was repeated 10 times, and standard deviation bars are plotted on each graph. A) Fraction of the original A20 & NIH3T3 DDG set that was recovered in the down-sampled cases. B) Fraction of the original A20 & NIH3T3 HVG set that was recovered in the down-sampled cases. C) Fraction of the original Zheng 9 DDG set that was recovered in the down-sampled cases. D) Fraction of the original Zheng 9 HVG set that was recovered in the down-sampled cases. (TIF) [file pcbi.1012386.s004.tif]

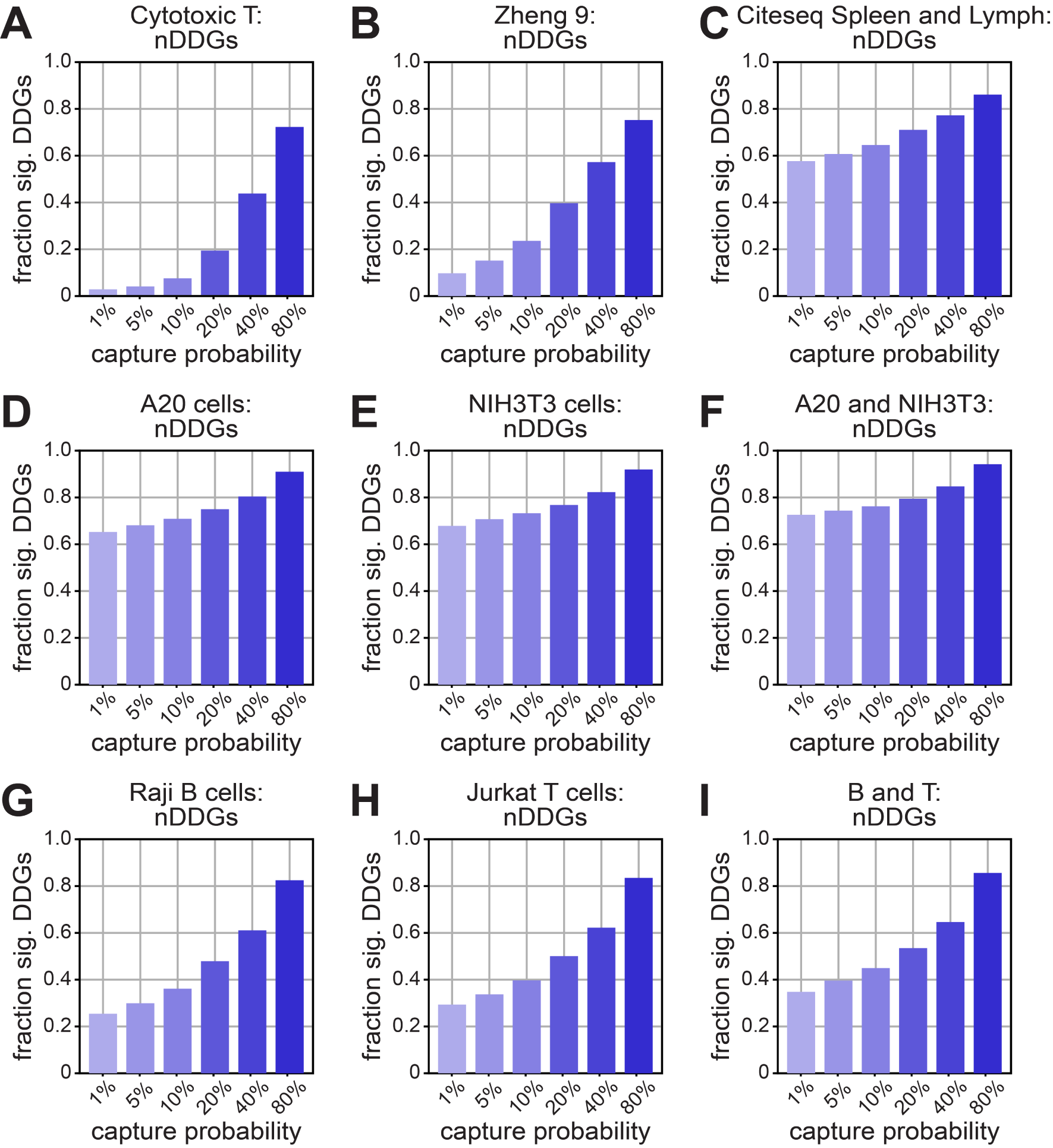

Supplement: S5 Fig — The effect of the capture probability parameter on DDG selection A-I) For each dataset, the capture probability parameter in the DDG model, pc, was titrated from 1% up to 80%. Bar graphs illustrate the number of DDGs identified at different pc, values for A) 10x scRNA-seq data generated from Cytotoxic T cells purified by FACs, B) 10x scRNA-seq data generated from the full set of Zheng 9 lymphocytes, C) Citeseq data generated from Spleen and Lymph cells, D) 10x scRNA-seq data generated from the A20 cell line, E) 10x scRNA-seq data generated from the NIH3T3 cell line, F) 10x scRNA-seq data generated from A20 and NIH3T3 cell lines, G) 10x scRNA-seq data generated from the Raji B cell line, H) 10x scRNA-seq data generated from the Jurkat T cell line, and I) 10x scRNA-seq data generated from the Raji B and the Jurkat T cell lines. (TIF) [file pcbi.1012386.s005.tif]

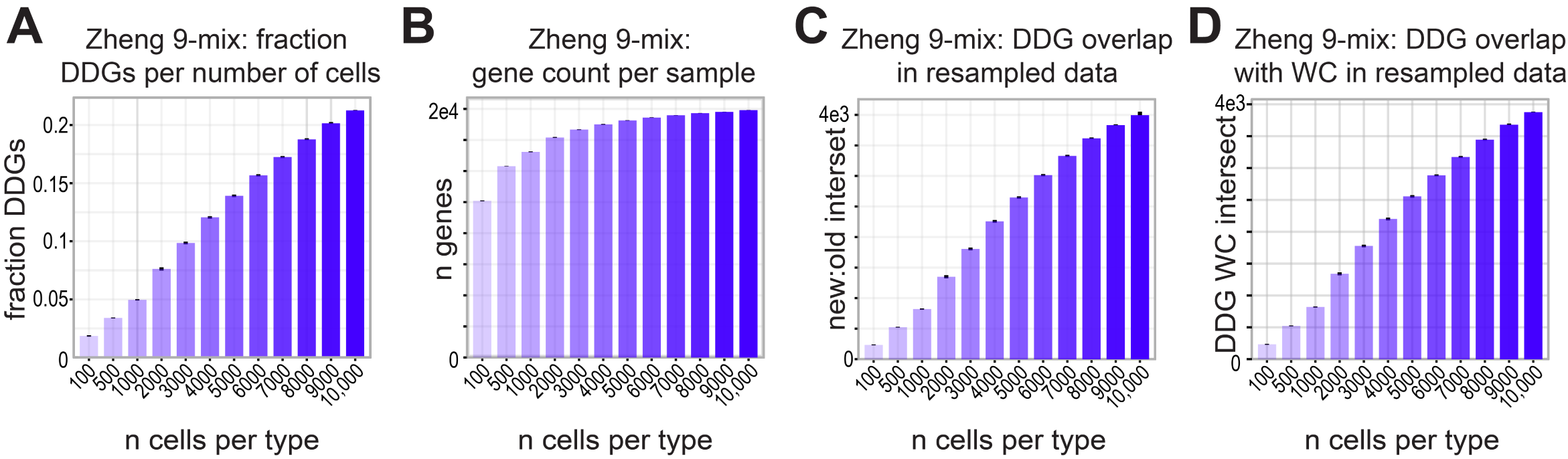

Supplement: S6 Fig — A-D) Empirical power estimations for the DDG method using the Zheng-9 lymphocyte mix, where a new set of DDGs was computed with increasing sample size. A) Mean number of DDGs as a function of increasing number of cells per cell type in the Zheng-9 lymphocyte mix. B) Mean total number of genes as a function of increasing cell number per sample. C) Mean overlap of new DDG sets with original DDG set calculated from the full, 5k cells per type Zheng-9 lymphocyte data. D) Mean overlap of new DDG sets with original Wilcoxon set of differentially expressed genes, as a function of number of cells per cell type. (TIF) [file pcbi.1012386.s006.tif]

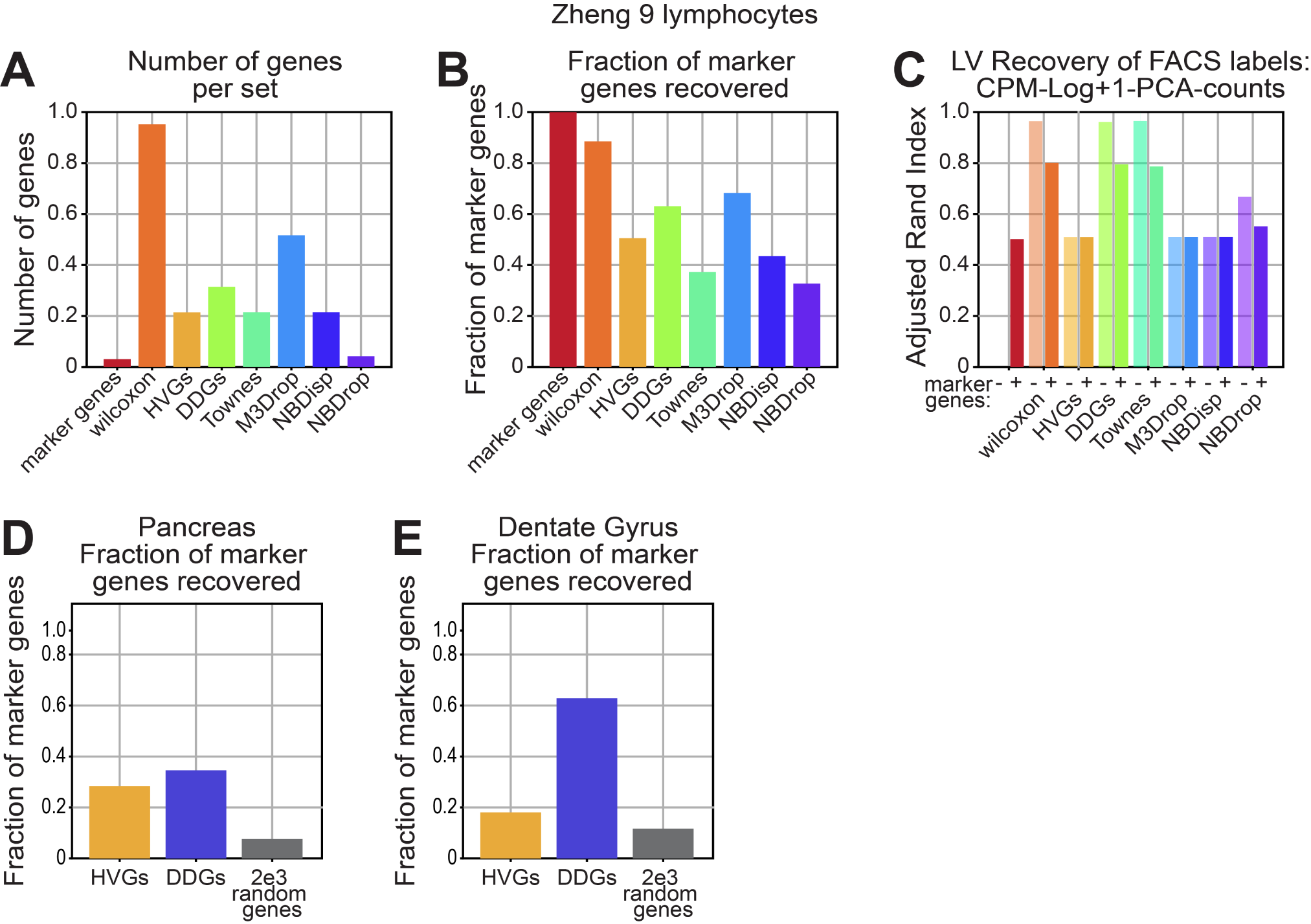

Supplement: S7 Fig — Cell type specific marker gene lists were downloaded from the Panglao database and used for this analysis. A) Number of genes in each feature set for the Zheng 9 lymphocytes. B) Fraction of marker gene set that is recovered in each feature set of the Zheng 9 lymphocytes. C) Quantification of FACS label recovery when the full set of marker genes were added to each feature set or when each feature set was used alone. Louvain clustering was performed across a titration of resolution parameters, cluster labels were compared to original FACS labels where T-cell lineages were merged into two super sets, and the highest adjusted rand index is plotted when log+1-CPM-PCA-counts were used as a basis. D) Fraction of marker gene set that is recovered in each feature set of the pancreas data. E) Fraction of marker gene set that is recovered in each feature set of the dentate gyrus data. (TIF) [file pcbi.1012386.s007.tif]

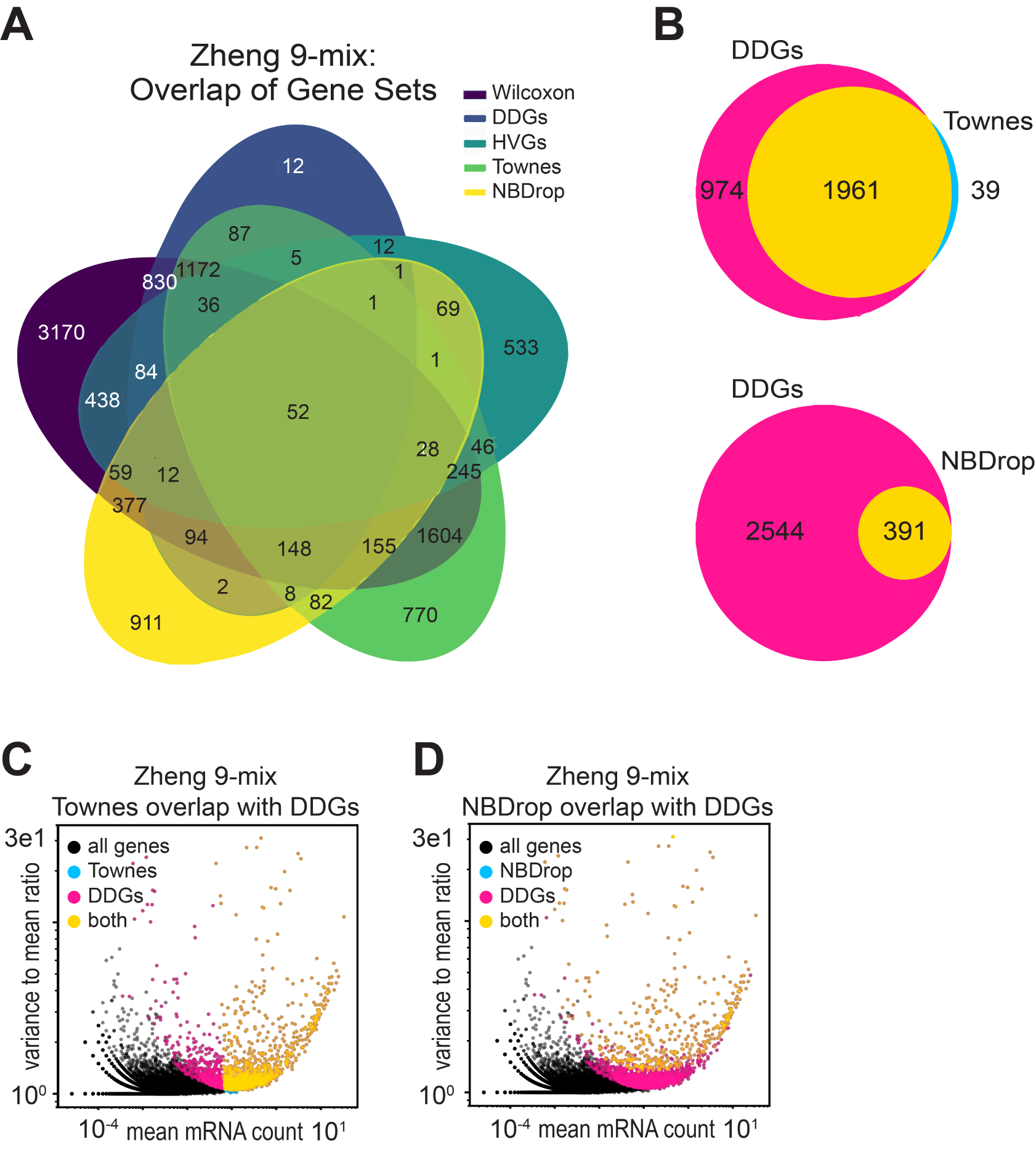

Supplement: S8 Fig — A) Venn diagram of set overlap for all feature sets calculated for the Zheng-9 lymphocyte mix. B) Venn diagrams of set overlap for the DDGs and the two other binomial based feature selection methods applied to the lymphocyte data. C,D) Scatter of the mean mRNA count versus dispersion per gene, colored by set membership for feature sets in the lymphocyte data. C) compares the Townes genes and DDGs while D) compares the NBDrop genes and DDGs. (TIF) [file pcbi.1012386.s008.tif]

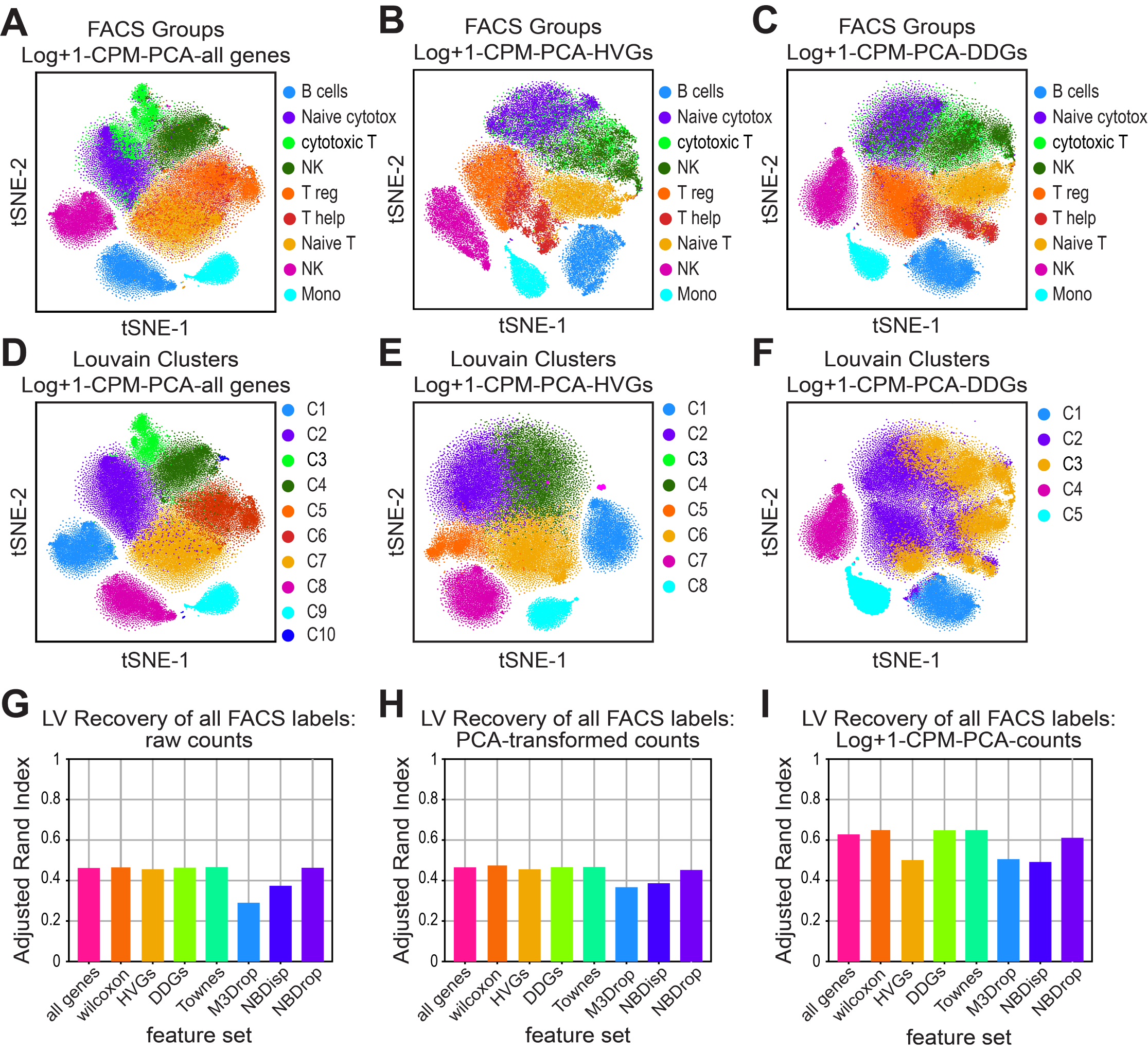

Supplement: S9 Fig — A-F) tSNE projections for dimensionality-reduced data from the Zheng-9 lymphocyte mix. Principal component analysis was used to reduce (A,D) all genes, (B,E) HVGs, or (C,F) DDGs after log+1 and CPM transforming the count data. Cells are either colored by original FACs-based set where T-cell lineages were merged into two super sets (A-C) or Louvain cluster membership (D-F). G-I) Quantification of FACS label recovery by various methods of dimensionality reduction across different feature sets. Louvain clustering was performed across a titration of resolution parameters, cluster labels were compared to original FACS labels where T-cell lineages were merged into two super sets, and the highest adjusted rand index is plotted when G) raw UMI counts, H) PCA-transformed counts, or F) log+1-CPM-PCA-counts were used as a basis. (TIF) [file pcbi.1012386.s009.tif]

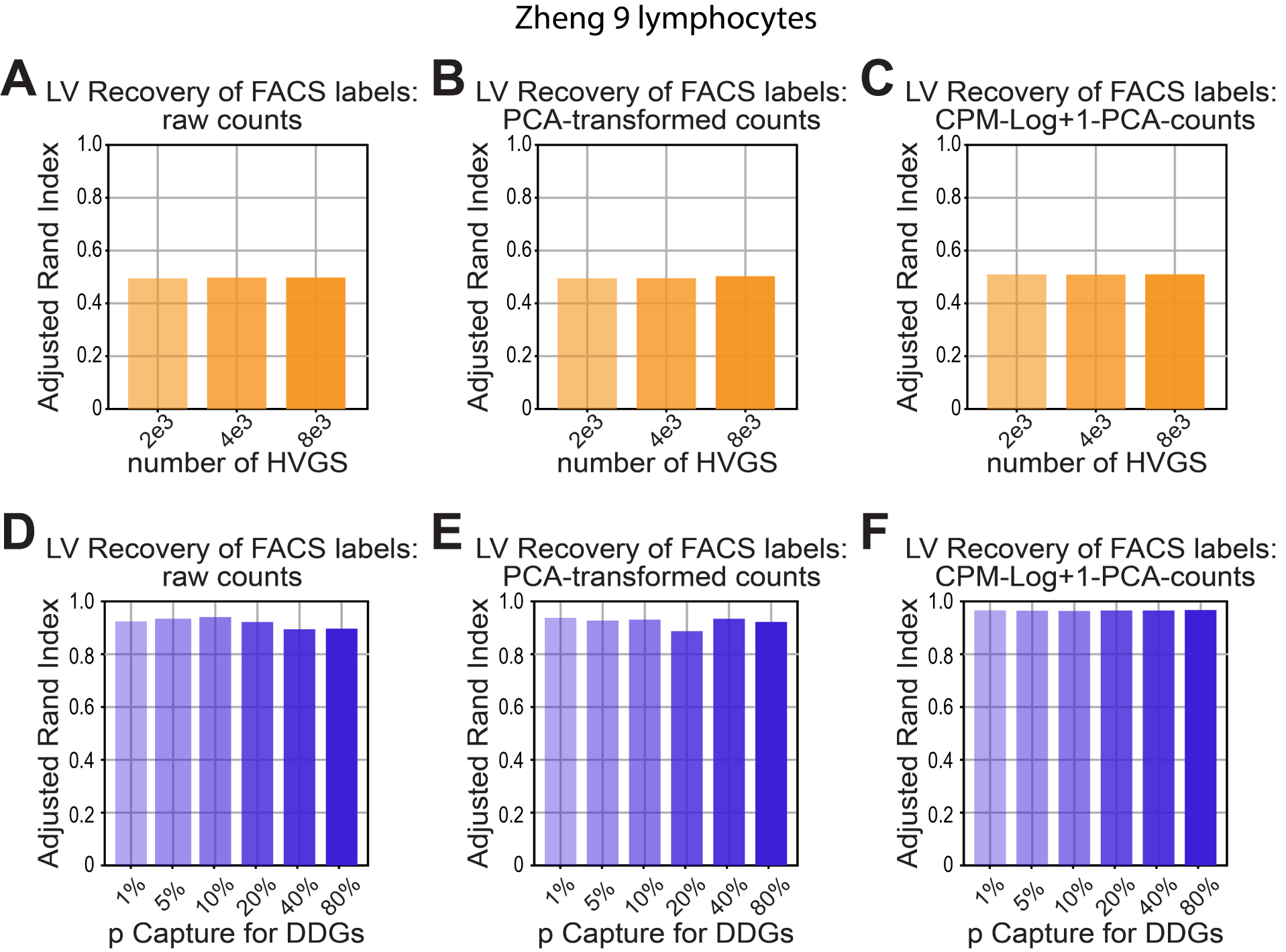

Supplement: S10 Fig — A-F) Quantification of FACS label recovery by various methods of dimensionality reduction across different feature sets. Louvain clustering was performed across a range of resolution parameters, cluster labels were compared to original FACS labels where T-cell lineages were merged into two supersets, and the highest adjusted rand index is plotted when A) raw UMI counts of HVGs, B) PCA-transformed HVG counts, C) log+1-CPM-PCA-HVG counts, D) raw UMI counts of DDGs, B) PCA-transformed DDG counts, C) log+1-CPM-PCA-DDG counts were used as a basis. (TIF) [file pcbi.1012386.s010.tif]

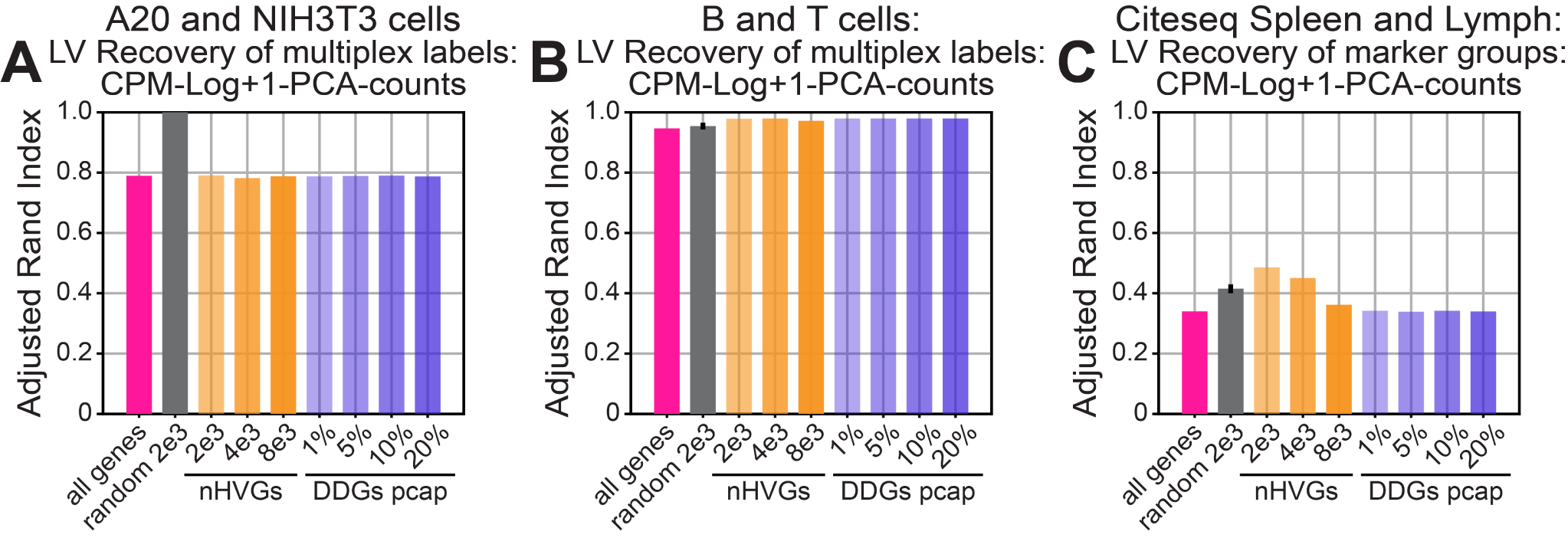

Supplement: S11 Fig — A-C) Quantification of orthogonal label recovery by various methods of dimensionality reduction across different feature sets. Louvain clustering was performed across a titration of resolution parameters, cluster labels were compared to cell-type labels, and the highest adjusted rand index is plotted when A) log+1-CPM-PCA counts from the multiplexed A20 and NIH3T3 cell lines, B) log+1-CPM-PCA counts from the multiplexed B and T cell lines, or C) log+1-CPM-PCA counts from the cell-surface protein gated Citeseq data, were used as a basis. (TIF) [file pcbi.1012386.s011.tif]

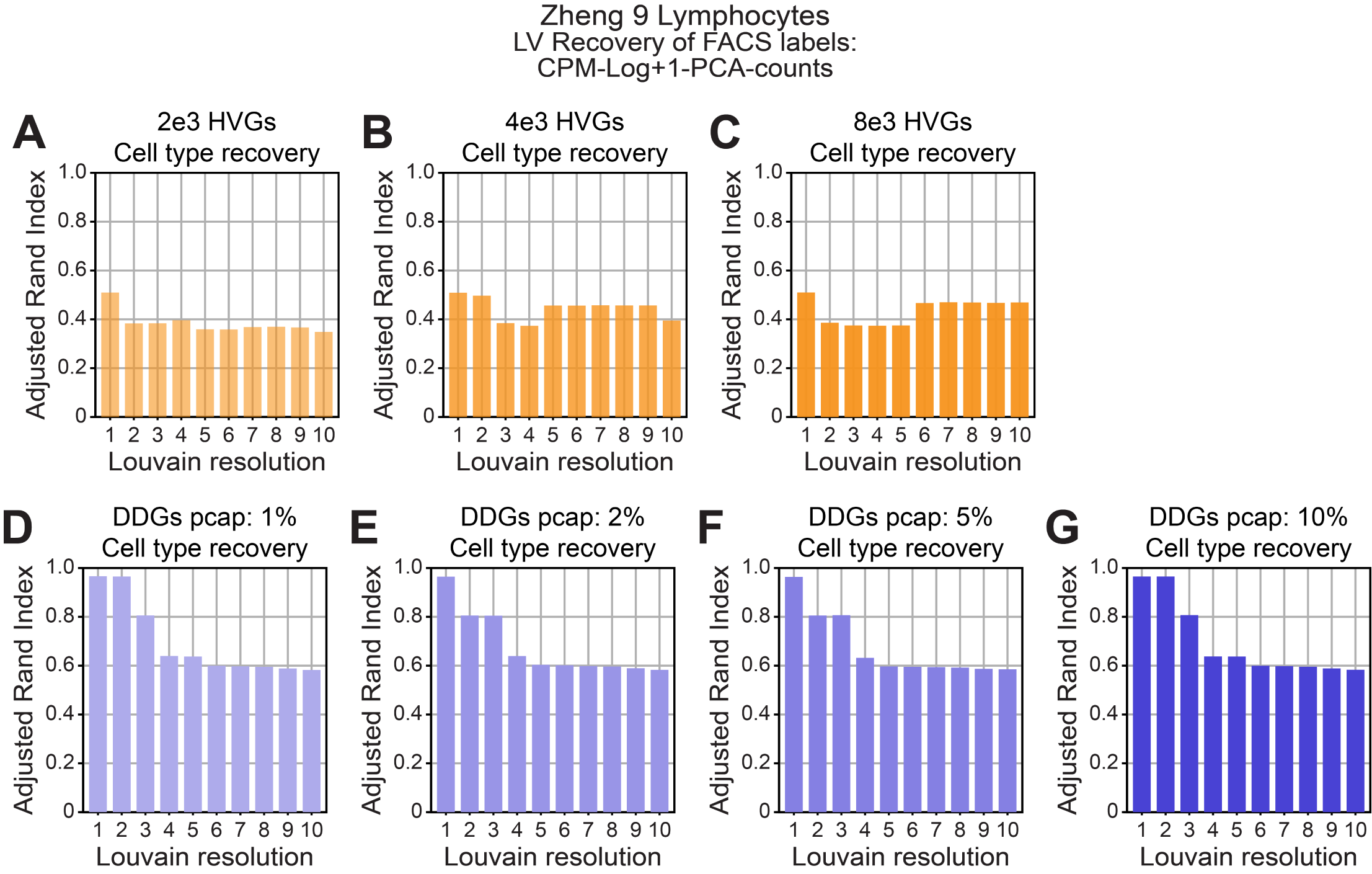

Supplement: S12 Fig — A-G) Quantification of FACS label recovery by various methods of dimensionality reduction across different feature sets and different Louvain clustering resolution parameters for the Zheng 9 lymphocyte data. Louvain clustering was performed across a titration of resolution parameters, cluster labels were compared to original FACS labels where T-cell lineages were merged into two super sets, and the adjusted rand index is plotted when A) log+1-CPM-PCA counts of 2e3 HVGs, B) 4e3 HVGs, or C) 8e3 HVGs, or log+1-CPM-PCA counts of DDGs identified with a pc of D) 1%, E) 2%, F) 5%, or G) 10% were used as a basis. (TIF) [file pcbi.1012386.s012.tif]

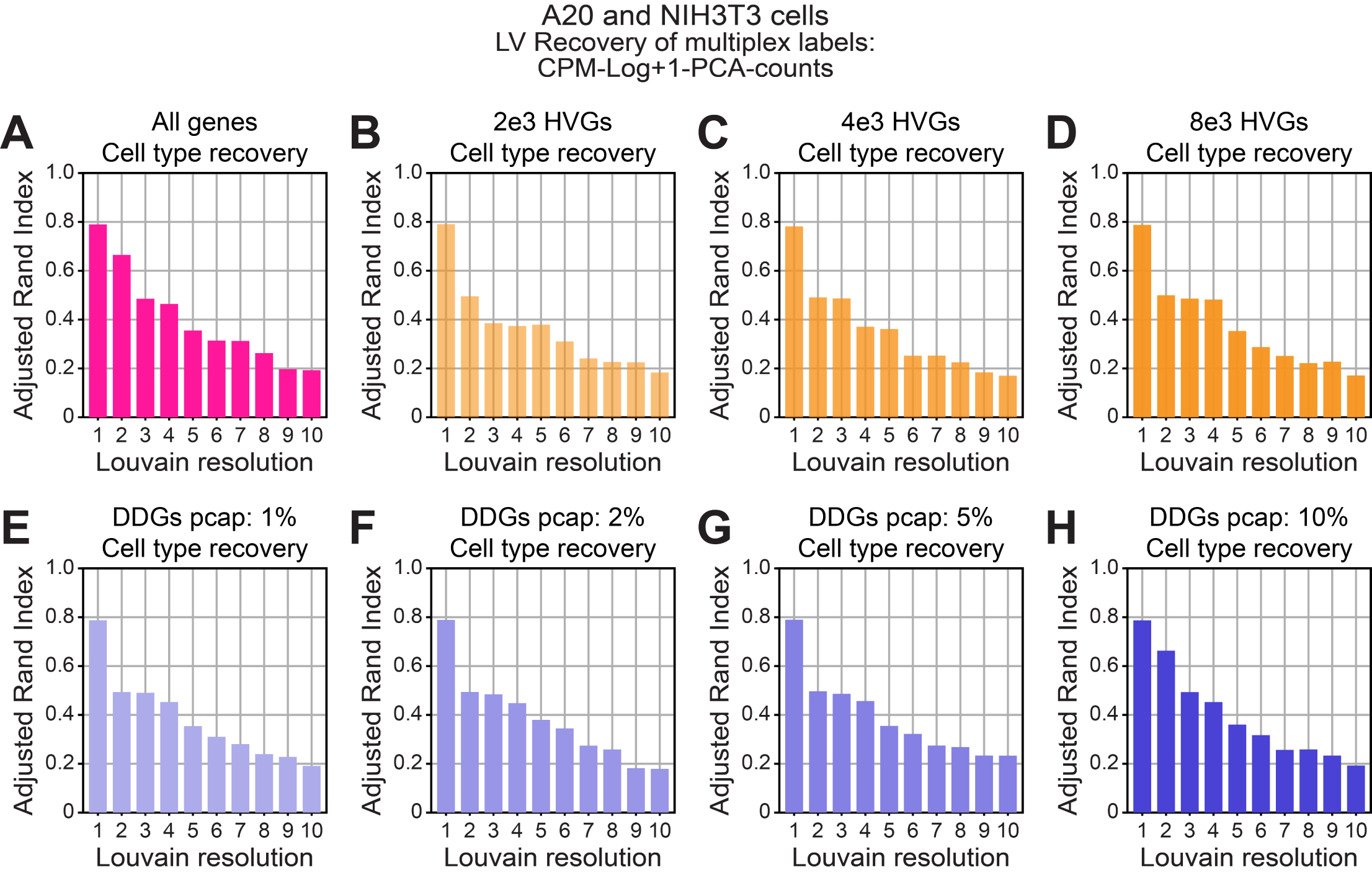

Supplement: S13 Fig — A-H) Quantification of multiplex label recovery by various methods of dimensionality reduction across different feature sets and different Louvain clustering resolution parameters for the multiplexed A20 and NIH3T3 cell line data. Louvain clustering was performed across a titration of resolution parameters, cluster labels were compared to original cell-type labels and the adjusted rand index is plotted when A) Log+1-CPM-PCA counts of all genes, B) log+1-CPM-PCA counts of 2e3 HVGs, C) 4e3 HVGs, or D) 8e3 HVGs, or log+1-CPM-PCA counts of DDGs identified with a pc of E) 1%, F) 2%, G) 5%, or H) 10% were used as a basis. (TIF) [file pcbi.1012386.s013.tif]

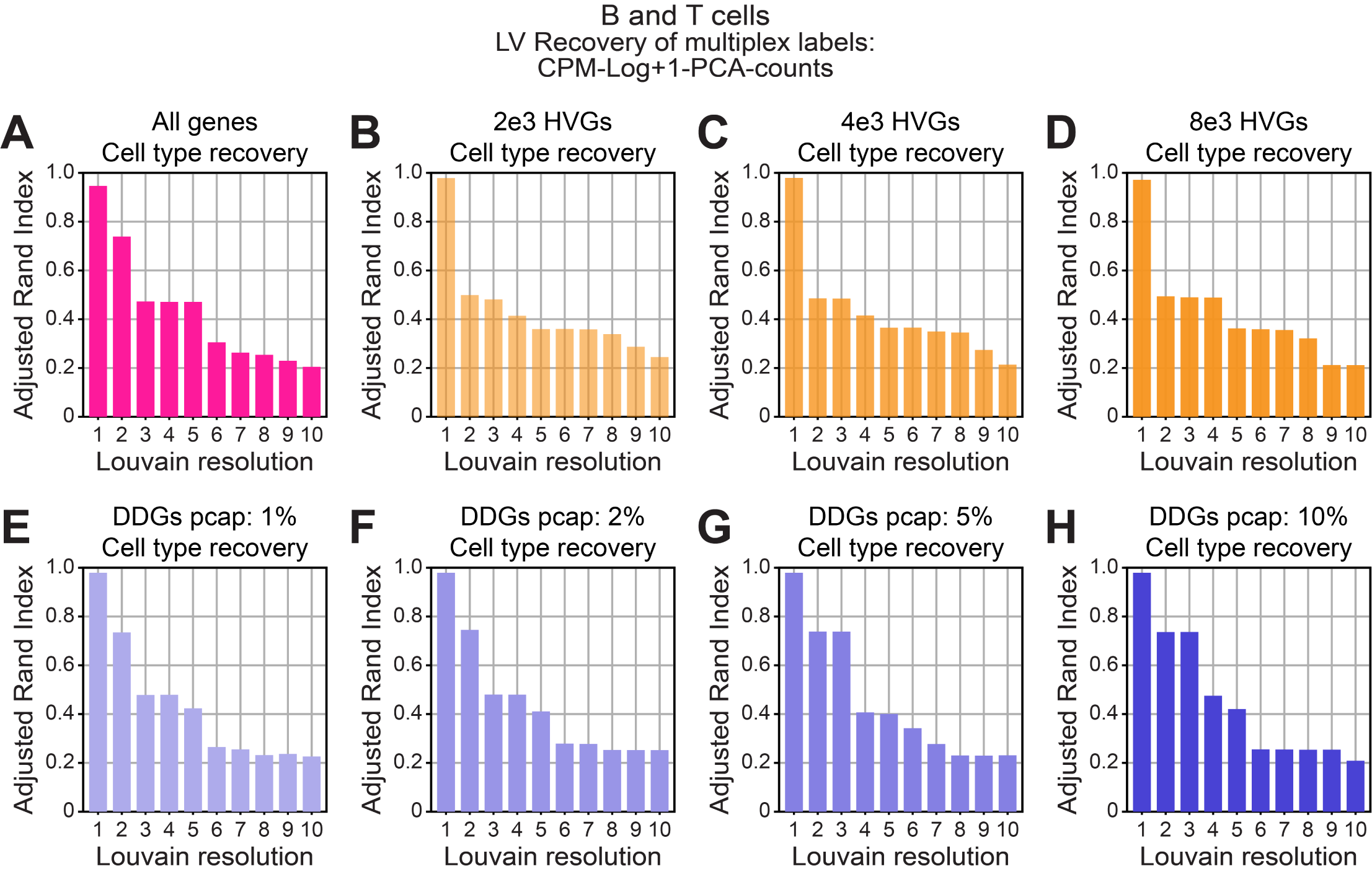

Supplement: S14 Fig — A-H) Quantification of multiplex label recovery by various methods of dimensionality reduction across different feature sets and different Louvain clustering resolution parameters for the multiplexed B and T cell line data. Louvain clustering was performed across a titration of resolution parameters, cluster labels were compared to original cell-type labels and the adjusted rand index is plotted when A) Log+1-CPM-PCA counts of all genes, B) log+1-CPM-PCA counts of 2e3 HVGs, C) 4e3 HVGs, or D) 8e3 HVGs, or log+1-CPM-PCA counts of DDGs identified with a pc of E) 1%, F) 2%, G) 5%, or H) 10% were used as a basis. (TIF) [file pcbi.1012386.s014.tif]

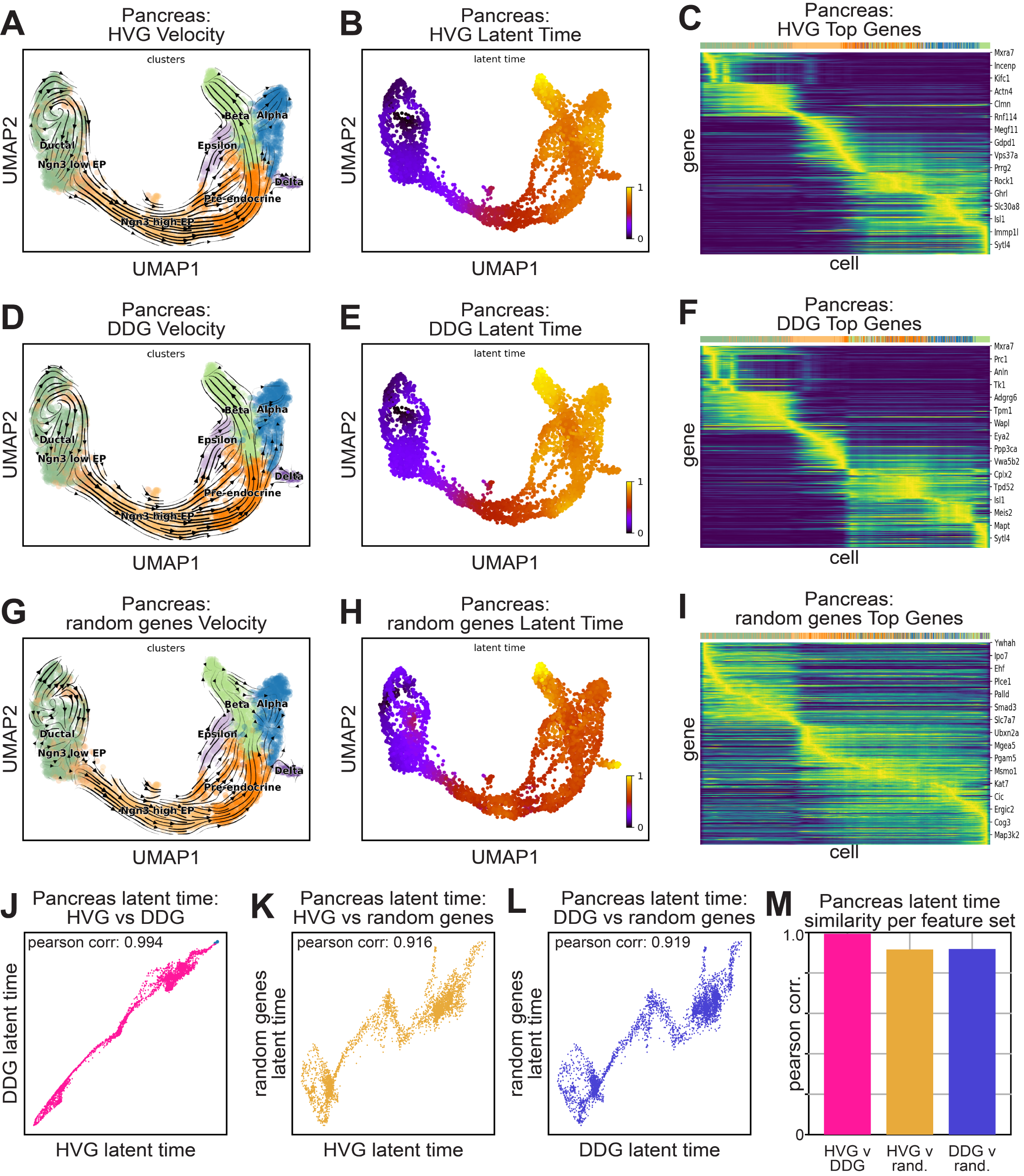

Supplement: S15 Fig — A-C) RNA velocity was performed using the scVelo software package and HVGs as the basis. A) UMAP projection of pancreas cells colored by HVG-based velocity and cluster membership. B) UMAP projection of pancreas cells colored by HVG-inferred latent time. C) Heatmap of gene counts of the 300 top genes contributing to the HVG-based latent time estimate, where each row represents a gene and each column represents individual cell that is ordered along the latent time axis. D-F) RNA velocity was performed using the scVelo software package and DDGs as the basis. D) UMAP projection of pancreas cells colored by DDG-based velocity and cluster membership. E) UMAP projection of pancreas cells colored by DDG-inferred latent time. F) Heatmap of gene counts of the 300 top genes contributing to the DDG-based latent time estimate, where each row represents a gene and each column represents individual cell that is ordered along the latent time axis. G-I) RNA velocity was performed using the scVelo software package and 2e3 random genes as the basis. G) UMAP projection of pancreas cells colored by random gene-based velocity and cluster membership. H) UMAP projection of pancreas cells colored by random gene-inferred latent time. I) Heatmap of gene counts of the 300 top genes contributing to the random gene-based latent time estimate, where each row represents a gene and each column represents an individual cell that is ordered along the latent time axis. J-L) Scatter plots of latent times assigned to each cell for J) HVGs and DDGs, K) HVGs and random genes, and L) DDGs and random genes. M) Pearson correlations of latent times inferred for each feature set compared. (TIF) [file pcbi.1012386.s015.tif]

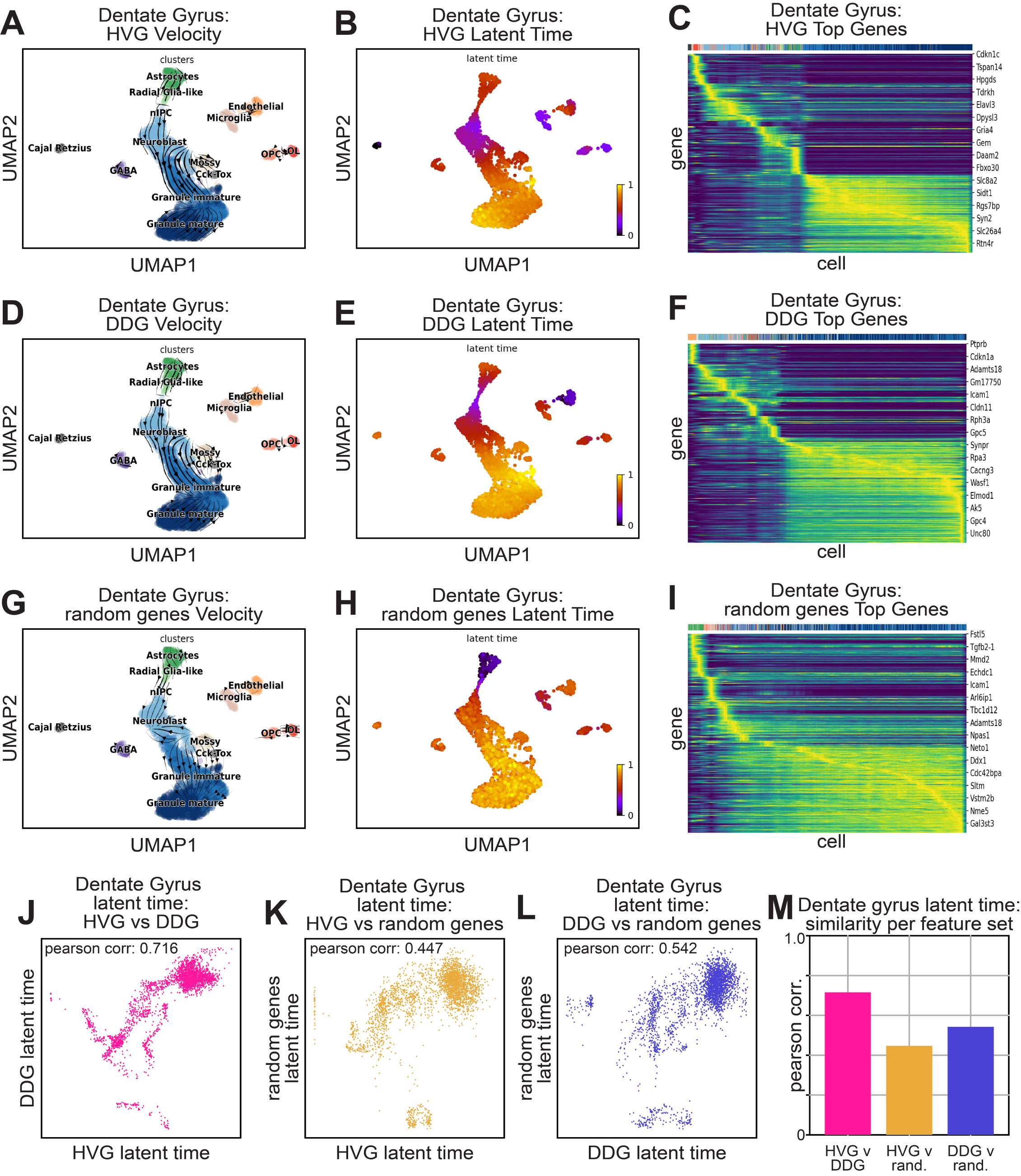

Supplement: S16 Fig — A-C) RNA velocity was performed using the scVelo software package and HVGs as the basis. A) UMAP projection of dentate gyrus cells colored by HVG-based velocity and cluster membership. B) UMAP projection of dentate gyrus cells colored by HVG-inferred latent time. C) Heatmap of gene counts of the 300 top genes contributing to the HVG-based latent time estimate, where each row represents a gene and each column represents individual cell that is ordered along the latent time axis. D-F) RNA velocity was performed using the scVelo software package and DDGs as the basis. D) UMAP projection of dentate gyrus cells colored by DDG-based velocity and cluster membership. E) UMAP projection of dentate gyrus cells colored by DDG-inferred latent time. F) Heatmap of gene counts of the 300 top genes contributing to the DDG-based latent time estimate, where each row represents a gene and each column represents individual cell that is ordered along the latent time axis. G-I) RNA velocity was performed using the scVelo software package and 2e3 random genes as the basis. G) UMAP projection of dentate gyrus cells colored by random gene-based velocity and cluster membership. H) UMAP projection of dentate gyrus cells colored by random gene-inferred latent time. I) Heatmap of gene counts of the 300 top genes contributing to the random gene-based latent time estimate, where each row represents a gene and each column represents an individual cell that is ordered along the latent time axis. J-L) Scatter plots of latent times assigned to each cell for J) HVGs and DDGs, K) HVGs and random genes, and L) DDGs and random genes. M) Pearson correlations of latent times inferred for each feature set compared. (TIF) [file pcbi.1012386.s016.tif]
